# Ethnic differences in depression and anxiety among adults with atopic eczema: Population‐based matched cohort studies within UK primary care

**DOI:** 10.1002/clt2.12348

**Published:** 2024-03-25

**Authors:** Elizabeth I. Adesanya, Alasdair Henderson, Joseph F. Hayes, Alexandra Lewin, Rohini Mathur, Amy Mulick, Caroline Morton, Catherine Smith, Sinéad M. Langan, Kathryn E. Mansfield

**Affiliations:** ^1^ Department of Non‐Communicable Disease Epidemiology London School of Hygiene & Tropical Medicine London UK; ^2^ Division of Psychiatry University College London London UK; ^3^ Department of Medical Statistics London School of Hygiene & Tropical Medicine London UK; ^4^ Centre for Primary Care Wolfson Institute of Population Health Queen Mary University of London London UK; ^5^ St John's Institute of Dermatology Guys and St Thomas' Foundation Trust and King's College London London UK

**Keywords:** anxiety, atopic eczema, depression, epidemiology, ethnicity

## Abstract

**Background:**

Evidence demonstrates that individuals with atopic eczema (eczema) have increased depression and anxiety; however, the role of ethnicity in these associations is poorly understood. We aimed to investigate whether associations between eczema and depression or anxiety differed between adults from white and minority ethnic groups in the UK.

**Methods:**

We used UK Clinical Practice Research Datalink GOLD to conduct matched cohort studies of adults (≥18 years) with ethnicity recorded in primary care electronic health records (April 2006‐January 2020). We matched (age, sex, practice) adults with eczema to up to five adults without. We used stratified Cox regression with an interaction between eczema and ethnicity, to estimate hazard ratios (HRs) for associations between eczema and incident depression and anxiety in individuals from white ethnic groups and a pooled minority ethnic group (adults from Black, South Asian, Mixed and Other groups).

**Results:**

We identified separate cohorts for depression (215,073 with eczema matched to 646,539 without) and anxiety (242,598 with eczema matched to 774,113 without). After adjusting for matching variables and potential confounders (age, sex, practice, deprivation, calendar period), we found strong evidence (*p* < 0.01) of ethnic differences in associations between eczema and depression (minority ethnic groups: HR = 1.33, 95% CI = 1.22,1.45; white ethnic groups: HR = 1.15, 95% CI = 1.12,1.17) and anxiety (minority ethnic groups: HR = 1.41, 95% CI = 1.28,1.55; white ethnic groups: HR = 1.17, 95% CI = 1.14,1.19).

**Conclusions:**

Adults with eczema from minority ethnic groups appear to be at increased depression and anxiety risk compared with their white counterparts. Culturally adapted mental health promotion and prevention strategies should be considered in individuals with eczema from minority ethnic groups.

## 1 INTRODUCTION

Atopic eczema (referred to as eczema throughout) is a common inflammatory skin disease (affecting between 2% and 10% of adults) associated with substantial morbidity and impaired quality of life.^1^ Depression and anxiety are common mental disorders associated with increased morbidity,^2^, ^3^ which can worsen prognosis and increase mortality in people with another medical condition.^2^ Worldwide, depressive disorders are the second, and anxiety disorders the eighth, leading causes of years lived with disability.^4^ In the UK, figures from the 2014 Adult Psychiatric Morbidity Survey suggested that 17% of adults experienced symptoms of depression or anxiety (e.g., somatic symptoms, fatigue, concentration and forgetfulness, depressive ideas, worry, sleep problems) in the week prior to being interviewed.^5^, ^6^

Considerable evidence demonstrates that people with eczema are at increased risk of depression and anxiety,^7^, ^8^, ^9^, ^10^, ^11^, ^12^, ^13^ including temporal evidence suggesting that eczema precedes depression and anxiety diagnosis.^11^, ^12^, ^13^ Reasons for associations between eczema and depression or anxiety are likely to be multifactorial and may include lifestyle factors (e.g., smoking, harmful alcohol use),^14^, ^15^, ^16^, ^17^, ^18^ disturbed sleep due to itch,^19^, ^20^ stigma due to visible skin disease,^21^, ^22^ and the inflammatory process itself.^23^

Ethnicity is a potentially important factor in the association between eczema and depression or anxiety, which has not been fully explored. Evidence suggests individuals from minority ethnic groups experience greater prevalence and incidence of eczema, depression, and anxiety compared to people from white ethnic groups.^24^, ^25^, ^26^ There are also potential mechanisms by which ethnicity affects associations between eczema and depression or anxiety including: (1) darker skin in individuals from minority ethnic groups delaying eczema diagnosis and treatment, and subsequently impacting mental health^24^; (2) dyspigmentation in individuals with darker skin exacerbating feelings of stigmatisation, further contributing to development of depression or anxiety^24^; (3) various beliefs in different cultures associated with skin diseases that can profoundly affect mental health^27^; and (4) barriers to health service access, deprivation, discrimination and racism, all of which disproportionately affect individuals from minority ethnic groups and can reinforce health inequalities and also negatively impact mental health.^28^, ^29^ Genetics may also partially explain ethnic differences in associations between eczema and depression or anxiety; however, other mechanisms described are likely to play a more substantial role.^30^

Previous longitudinal studies have either not investigated the role of ethnicity in the associations between eczema and depression or anxiety, or only done so in sensitivity analyses.^8^, ^11^, ^12^, ^13^ It is therefore unknown whether depression or anxiety risk in people with eczema varies by ethnic group. Given the paucity of previous research, and the health inequalities experienced by those from minority ethnic groups, it is important to identify whether associations between eczema and depression or anxiety differ between ethnic groups.

We undertook two matched cohort studies using primary care electronic health record data to investigate whether associations between eczema and incident depression or anxiety differed between adults from white ethnic groups and a pooled minority ethnic group (including individuals from Black, South Asian, Mixed, and Other ethnicities). We also explored whether there were differences in eczema severity between individuals from white and minority ethnic groups, and whether associations varied with eczema severity.

## 2 METHODS

### 2.1 Study design and setting

We conducted matched cohort studies (April 1, 2006, to January 31, 2020), using primary care electronic health record data from the UK Clinical Practice Research Datalink (CPRD GOLD). CPRD is an ongoing, nationwide database of routinely collected primary care records including information on demographics, diagnoses, symptoms, and prescriptions.^31^ Morbidity code lists and all analytic codes used in this study are available for download.^32^

### 2.2 Study population

All adults (≥18 years) with complete ethnicity data and at least 1 year of registration with a general practice meeting CPRD quality‐control standards during the study period were eligible for inclusion. We matched (without replacement) each adult with eczema (on age [±5 years], sex, and practice) with up to five adults without eczema in calendar date order (Appendix S1). We did not match ethnicity to prevent introducing selection bias. Adults with eczema were identified using a previously validated definition requiring records of at least one eczema diagnostic code, and at least two eczema therapies (e.g., phototherapy, prescriptions for topical or oral drugs) recorded on separate days.^33^ We identified two matched cohorts – one to investigate depression (depression cohort) and the other to investigate anxiety (anxiety cohort) (although individuals included in the cohorts only varied based on exclusion due to previous anxiety/depression/SMI diagnosis). In both cohorts, we excluded individuals with relevant mental health conditions prior to cohort entry (e.g., exclusion of those with previous depression in the depression cohort; and previous anxiety in the anxiety cohort; concurrent depression and anxiety, and severe mental illness – i.e., schizophrenia, bipolar disorder, and other psychoses – in both depression and anxiety cohorts).

We followed individuals with eczema from the latest of (index date): study start (April 1, 2006); date they met our eczema definition; 1 year after the date of registration with their practice; date their practice met CPRD quality‐control standards; or their 18th birthday. Follow‐up for individuals without eczema began on the same date as their matched individual with eczema. Follow‐up ended at the earliest of: depression or anxiety diagnosis or symptom recording (depending on the outcome under investigation); diagnosis of severe mental illness (suggests an alternative cause for the depression or anxiety); study end (January 31, 2020); end of registration with practice; practice no longer contributing data to CPRD; eczema diagnosis (for adults without); or death. Individuals without eczema with an eczema diagnostic code during follow‐up were censored from the comparison cohort and become eligible for inclusion in the eczema cohort.

### 2.3 Outcomes

We considered depression and anxiety as separate outcomes. We identified depression and anxiety based on the earliest record of a diagnostic or symptom morbidity code recorded in primary care as evidence suggests that GPs record depression and anxiety symptoms as well as diagnoses.^34^, ^35^, ^36^ We considered broader definitions of depression and anxiety in sensitivity analyses (Table 1).

**TABLE 1:** Description and rationale of sensitivity analyses.

Description of sensitivity analyses	Rationale
Repeating the main analysis using alternative code lists to identify depression and anxiety outcomes (including broader codes and symptom codes)	To explore the sensitivity of the results due to the definitions of the depression and anxiety outcomes
Restricting cohort entry to individuals with at least one consultation with their GP in the year before cohort entry.	To exclude individuals who are practice non‐attenders. There may be differential recording of exposure, covariates and outcomes among practice attenders and non‐attenders. For example, practice non‐attenders may be more likely to have missing smoking or BMI data.
Repeating the main analysis after removing censoring at the time of an alternative diagnoses that may also represent the outcome of interest (i.e., severe mental illness).	To avoid potentially informative censoring of outcomes by severe mental illness.
Repeating the main analysis using a redefined cohort of adults entering from 1 April 2006 that are eligible for linkage with HES and have complete ethnicity data.	To explore the sensitivity of our results to the definition of our study population and examine whether the study population of the main analysis is susceptible to selection bias. The main study population included only individuals with complete ethnicity data who are likely to be different from those with missing ethnicity data, which may introduce selection bias. Previous work has shown that combining CPRD and HES increases the completeness of ethnicity data.
Repeating the main analysis using a redefined cohort of adults entering from 1 April 2006. Missing ethnicity data was imputed using multiple imputation.	To explore the sensitivity of our results to the definition of our study population and examine whether the study population of the main analysis is susceptible to selection bias.
Repeating the main analysis using less strict definitions for sleep problems (main analysis code list includes Zolpidem and Zopiclone which are only prescribed for sleep problems, sensitivity analysis code list expanded to include prescriptions for benzodiazepines, melatonin, and other drugs).	To explore whether including broader drugs that are prescribed for conditions other than sleep disturbances further mediates the association between atopic eczema and severe mental illness.

### 2.4 Covariates

We identified ethnicity using a previously validated algorithm using primary care records to classify individuals into five ethnic groups: White, Black, South Asian, Mixed, and Other.^37^ Due to limited statistical power, we pooled individuals from Black, South Asian, Mixed and Other ethnic groups into a ‘minority ethnic’ group. We used this grouping to investigate whether associations between eczema and depression or anxiety differed between individuals from white and minority ethnic groups.

We used a directed acyclic graph (DAG) and a systematic review to inform covariate selection (Appendix S2).^38^, ^39^ We matched age, sex, and practice (as an indirect means of accounting for differences in coding practice, rural/urban location, and socioeconomic deprivation). We considered age, sex, calendar period (2006–2010, 2011–2015, 2016–2020) and deprivation (quintiles of individual‐level Carstairs Index used when available, and practice‐level Carstairs if not) as potential confounders.^40^ We considered the following variables as potential mediators (captured on or before index date): comorbidity burden (captured using Charlson Comorbidity Index [CCI]),^41^ comorbid asthma (identified using primary care morbidity coding), body mass index (BMI), smoking status, harmful alcohol use, sleep problems and high‐dose glucocorticoid use (Appendix S3 includes details of covariate definitions).

### 2.5 Statistical analyses

#### 2.5.1 Main analysis

We initially described the characteristics of adults with and without eczema. We used Cox regression, stratified by matched set with current age as the underlying timescale, to estimate hazard ratios (HRs) and 95% confidence intervals (CIs) for associations between eczema and depression or anxiety in individuals from white and minority ethnic groups.

We initially constructed minimally adjusted models including an interaction term between eczema and ethnicity, implicitly adjusting for age (through underlying timescale) and matching variables (age, sex, practice) by stratifying on matched set. We then followed with sequential models: (1) additionally adjusted for potential confounders (deprivation, calendar period) to investigate the total associations through eczema and other potential mediating variables of eczema on depression/anxiety in white and minority ethnic groups; and (2) further adjusted for factors potentially on the causal pathway that may mediate associations between eczema and depression/anxiety (comorbidity burden, comorbid asthma, BMI, smoking status, harmful alcohol use, sleep problems, high‐dose glucocorticoid use). We performed likelihood ratio tests to assess whether there is an interaction between eczema and ethnicity. We assessed proportional hazard assumptions using Schoenfeld residuals (Appendix S4). All data were managed and analysed using Stata V17 (StataCorp).

We repeated our main analysis in a series of sensitivity analyses to assess the robustness of our findings (Tables 1, S1–S4, Appendix S5).

#### 2.5.2 Secondary analyses

We classified adults with eczema as having mild, moderate, or severe disease using a previously developed, time‐updated definition based on primary care morbidity coding and prescriptions (Appendix S6).^42^ To explore whether there were differences in eczema severity (based on primary care recording) between individuals from white and minority ethnic groups, we described the proportions of total follow‐up adults with eczema in each ethnic group (white or minority ethnic) spent at each level of eczema severity. We also estimated HRs and 95% Cis for associations between eczema severity and depression or anxiety in individuals from white and minority ethnic groups.

## 3 RESULTS

**FIGURE 1:**
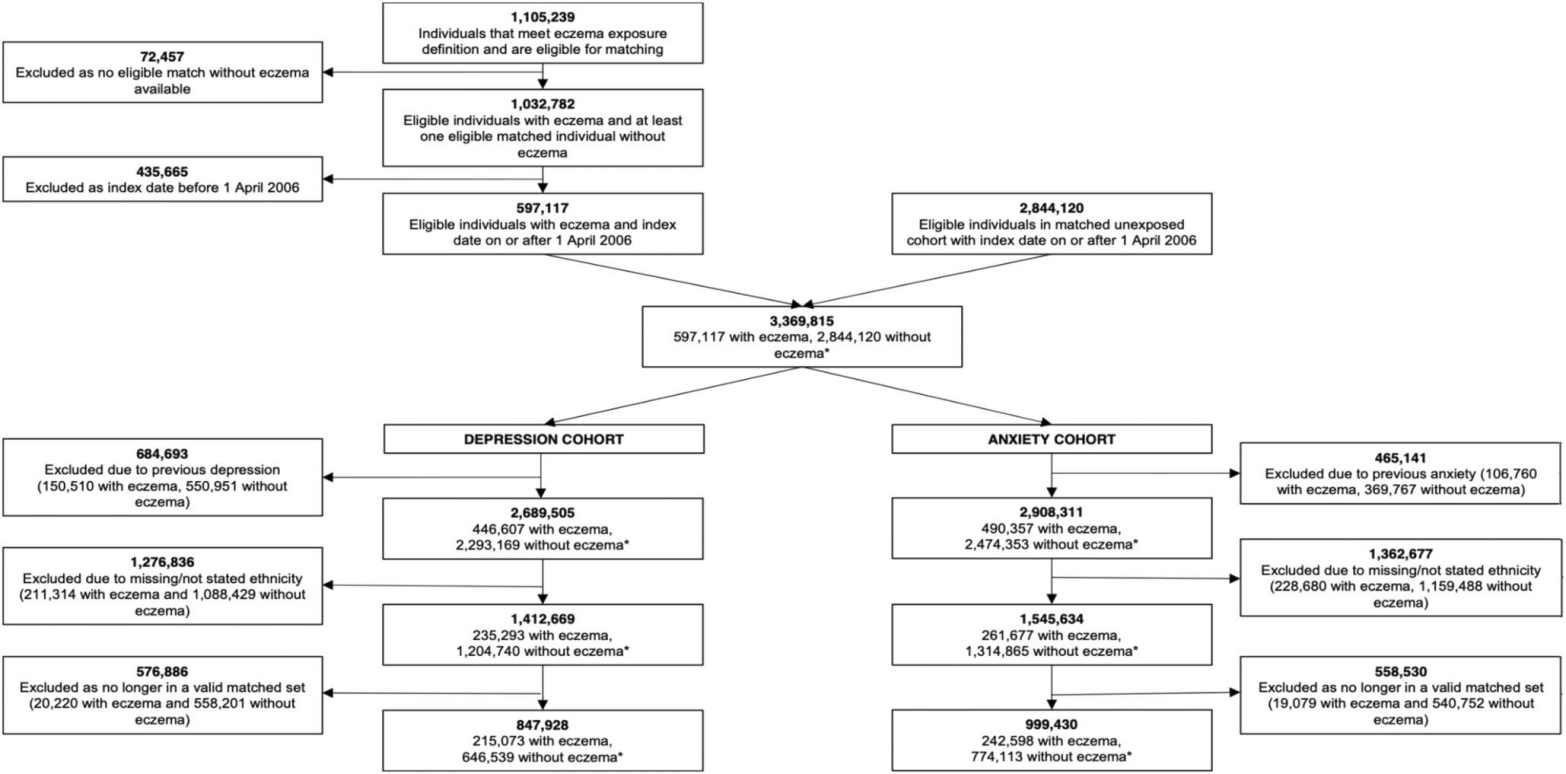
Flowchart illustrating the identification of participants in depression and anxiety cohorts. *Numbers of people with and without atopic eczema do not sum to the total number of individuals included in each cohort. Individuals with atopic eczema could be included in the matched comparison cohort up to the date of their first atopic eczema diagnosis.

We identified 597,117 adults with eczema matched to 2,844,120 adults without (Figure 1). After excluding individuals with missing ethnicity data, those with the outcome of interest or severe mental illness on or before start of follow‐up, and those who no longer remained in valid matched sets (i.e., matched set including at least one exposed and one unexposed individual): the depression cohort included 215,073 adults with eczema matched to 646,539 without; and the anxiety cohort included 242,598 adults with eczema matched to 774,113 without. Individuals with missing ethnicity data were more likely to be younger, male, and have missing BMI and smoking status (Table S5).

In those with eczema, median follow‐up was between 3.2 and 3.3 years (depression cohort 3.2 [IQR:1.3–5.9], anxiety cohort 3.3 [IQR:1.4–6.0]), and in those without eczema between 2.8 and 2.9 years (depression cohort 2.8 [IQR:1.2–5.5], anxiety cohort 2.9 [IQR:1.2–5.6]). In both cohorts, people with and without eczema had broadly similar age, sex, deprivation, and ethnicity (Tables 2, S6). Fewer adults with eczema had missing BMI and smoking status, and those with missing data were more likely to be younger and male compared with those with complete data (Tables S7 and S8).

**TABLE 2:** Characteristics of depression and anxiety cohorts at cohort entry.

	Depression cohort		Anxiety cohort	
	With atopic eczema	Without atopic eczema	With atopic eczema	Without atopic eczema
	*n* = 215,073	*n* = 646,539	*n* = 242,598	*n* = 774,113
Follow‐up^a^				
Total person‐years	845,534	2,381,779	970,230	2,898,227
Median (IQR) duration of follow‐up (years)	3.2 (1.3–5.9)	2.8 (1.2–5.5)	3.3 (1.4–6.0)	2.9 (1.2–5.6)
Sex				
Female (%)	119,149 (55.4%)	354,208 (54.8%)	138,964 (57.3%)	446,169 (57.6%)
Age (years)^b^				
18–29	83,066 (38.6%)	267,060 (41.3%)	88,755 (36.6%)	297,014 (38.4%)
30–39	36,715 (17.1%)	113,881 (17.6%)	42,298 (17.4%)	140,665 (18.2%)
40–49	25,545 (11.9%)	70,699 (10.9%)	31,182 (12.9%)	94,977 (12.3%)
50–59	21,605 (10.0%)	59,361 (9.2%)	26,090 (10.8%)	78,533 (10.1%)
60–69	21,999 (10.2%)	63,035 (9.7%)	25,257 (10.4%)	77,543 (10.0%)
70+	26,143 (12.2%)	72,503 (11.2%)	29,016 (12.0%)	85,381 (11.0%)
Ethnicity				
White	183,612 (85.4%)	548,100 (84.8%)	208,462 (85.9%)	661,005 (85.4%)
Minority ethnic	31,461 (14.6%)	98,439 (15.2%)	34,136 (14.1%)	113,108 (14.6%)
Quintiles of Carstairs deprivation index^c^				
1—Least deprived	40,005 (18.6%)	112,671 (17.4%)	44,123 (18.2%)	132,325 (17.1%)
2	42,331 (19.7%)	127,095 (19.7%)	46,993 (19.4%)	149,716 (19.3%)
3	44,016 (20.5%)	129,585 (20.0%)	49,443 (20.4%)	154,764 (20.0%)
4	45,293 (21.1%)	138,247 (21.4%)	52,153 (21.5%)	168,516 (21.8%)
5—Most deprived	40,855 (19.0%)	131,872 (20.4%)	47,062 (19.4%)	160,670 (20.8%)
Missing	2573 (1.2%)	7069 (1.1%)	2824 (1.2%)	8122 (1.0%)
Body mass index (kg/m^2^)^d^				
Underweight (<18.5)	5583 (2.6%)	18,965 (2.9%)	6156 (2.5%)	22,161 (2.9%)
Normal (18.5–24.9)	78,647 (36.6%)	233,286 (36.1%)	87,042 (35.9%)	276,952 (35.8%)
Overweight (25–29.9)	57,766 (26.9%)	163,116 (25.2%)	65,734 (27.1%)	198,662 (25.7%)
Obese (30+)	38,849 (18.1%)	105,050 (16.2%)	47,546 (19.6%)	136,952 (17.7%)
Missing	34,228 (15.9%)	126,122 (19.5%)	36,120 (14.9%)	139,386 (18.0%)
Smoking status^d^				
Non‐smoker	115,457 (53.7%)	346,669 (53.6%)	124,789 (51.4%)	399,346 (51.6%)
Current or ex‐smoker	95,696 (44.5%)	278,407 (43.1%)	113,859 (46.9%)	352,189 (45.5%)
Missing	3920 (1.8%)	21,463 (3.3%)	3950 (1.6%)	22,578 (2.9%)
Charlson comorbidity index^d^				
Low (0)	134,391 (62.5%)	479,693 (74.2%)	149,031 (61.4%)	565,797 (73.1%)
Moderate (1–2)	71,488 (33.2%)	139,122 (21.5%)	82,401 (34.0%)	172,496 (22.3%)
Severe (3 or more)	9194 (4.3%)	27,724 (4.3%)	11,166 (4.6%)	35,820 (4.6%)
Asthma (%)^d^	54,774 (25.5%)	82,629 (12.8%)	63,142 (26.0%)	102,633 (13.3%)
Harmful alcohol use (%)^d^	15,943 (7.4%)	40,279 (6.2%)	19,740 (8.1%)	53,118 (6.9%)
Problems with sleep (%)^d^	37,355 (17.4%)	66,682 (10.3%)	47,582 (19.6%)	93,110 (12.0%)

*Note*: Values are numbers (percentages) unless otherwise stated. Individuals can contribute data as both atopic eczema exposed and unexposed. Therefore, numbers of exposed/unexposed do not total the whole cohort, as individuals may be included in more than one column.

Abbreviation: IQR: Interquartile range.

^a^ Follow‐up based on censoring at the earliest of: death, no longer registered with practice, practice no longer contributing to CPRD, or depression or anxiety diagnosis, diagnosis that suggests an alternative cause of the depression or anxiety outcome (severe mental illness).

^b^ Age at index date.

^c^ Carstairs deprivation index based on practice‐level data (from 2011).

^d^ Based on records closest to index date.

### 3.1 Main analyses

We found strong evidence (*p* < 0.01) that associations between eczema and depression or anxiety varied by ethnic group (Table 3). After implicitly adjusting for matching variables (age, sex, practice) and adjusting for potential confounders (calendar period, deprivation), we found that people with eczema had increased hazards for depression (HR = 1.17, 95% CI = 1.14,1.19) and anxiety (HR = 1.19, 95% CI = 1.16,1.21) compared with matched comparators (Table S9). When stratified by ethnicity, hazards for depression (minority ethnic groups: HR = 1.33, 95% CI = 1.22,1.45; white ethnic groups: HR = 1.15, 95% CI = 1.12,1.17) and anxiety (minority ethnic groups: HR = 1.41, 95% CI = 1.28,1.55; white ethnic groups: HR = 1.17, 95% CI = 1.14,1.19) were higher in adults from minority ethnic groups than adults from white ethnic groups.

**TABLE 3:** HRs (95% CI)^a^ for the association between atopic eczema and depression or anxiety stratified by ethnic group.

Cohort	Minimally adjusted^c^			Further adjusted for potential confounders^d^			Additionally adjusted for potential mediators^e^		
	Number	Events/PYAR	HR (95% CI)	Number	Events/PYAR	HR (95% CI)	Number	Events/PYAR	HR (95% CI)
Depression									
White									
Without atopic eczema	548,100	42,656/2,083,588	1 (reference)	541,478	41,994/2,054,242	1 (reference)	385,813	31,151/1,542,760	1 (reference)
With atopic eczema	183,612	17,555/735,495	1.15 (1.12,1.17)	181,173	17,246/724,343	1.15 (1.12,1.17)	145,302	14,436/604,767	1.05 (1.03,1.08)
Minority ethnic									
Without atopic eczema	98,439	3847/298,191	1 (reference)	97,992	3839/296,708	1 (reference)	69,137	2989/222,425	1 (reference)
With atopic eczema	31,461	1926/110,039	1.32 (1.21,1.44)	31,327	1919/109,442	1.33 (1.22, 1.45)	25,478	1665/93,033	1.14 (1.02,1.26)
Anxiety									
White									
Without atopic eczema	661,005	38,443/2,550,372	1 (reference)	653,373	37,781/2,515,225	1 (reference)	478,957	29,019/1,934,703	1 (reference)
With atopic eczema	208,462	15,384/848,896	1.17 (1.14,1.19)	205,779	15,080/836,483	1.17 (1.14,1.19)	168,344	12,849/710,076	1.07 (1.04,1.09)
Minority ethnic									
Without atopic eczema	113,108	3227/347,855	1 (reference)	112,618	3215/346,231	1 (reference)	81,361	2469/265,107	1 (reference)
With atopic eczema	34,136	1509/121,334	1.40 (1.27,1.54)	33,995	1501/120,702	1.41 (1.28,1.55)	27,963	1322/103,522	1.22 (1.09, 1.37)

*Note*: Fitted to adults with complete data for all variables included in each model and from valid matched sets^b^. *p* values for interaction by ethnicity is *p* < 0.01 for all models and outcomes.

Abbreviations: CI, Confidence Interval; HR, Hazard Ratio; PYAR, Person years at risk.

^a^ Estimated hazard ratios from Cox regression with current age as underlying timescale, stratified by matched set (matched on age at cohort entry, sex, general practice, and date at cohort entry).

^b^ Matched sets including one individual with atopic eczema and at least one matched comparator without.

^c^ Adjusted for matching variables (age, sex, practice).

^d^ Minimally adjusted model further adjusted for calendar period and deprivation (using quintiles of Carstairs deprivation index [using 2011 census data]).

^e^ Cohort is further adjusted for comorbidity burden (using the Charlson comorbidity index), comorbid asthma, sleep problems, smoking status, high dose glucocorticoid use, harmful alcohol use and body mass index.

After additionally adjusting for potential mediators (CCI, comorbid asthma, harmful alcohol use, smoking status, BMI, sleep problems, high‐dose glucocorticoid use), estimates of associations between eczema and depression (minority ethnic groups: HR = 1.14, 95% CI = 1.02,1.26; white ethnic groups: HR = 1.05, 95% CI = 1.03,1.08) and anxiety (minority ethnic groups: HR = 1.22, 95% CI = 1.09,1.37; white ethnic groups: HR = 1.07, 95% CI = 1.04,1.09) remained higher among adults from minority ethnic groups.

In all sensitivity analyses (Tables S1–S4, Appendix S5), we saw broadly similar results to the main analysis with more elevated hazards of depression and anxiety among adults from minority ethnic groups compared with adults from white ethnic groups.

### 3.2 Secondary analyses

**FIGURE 2:**
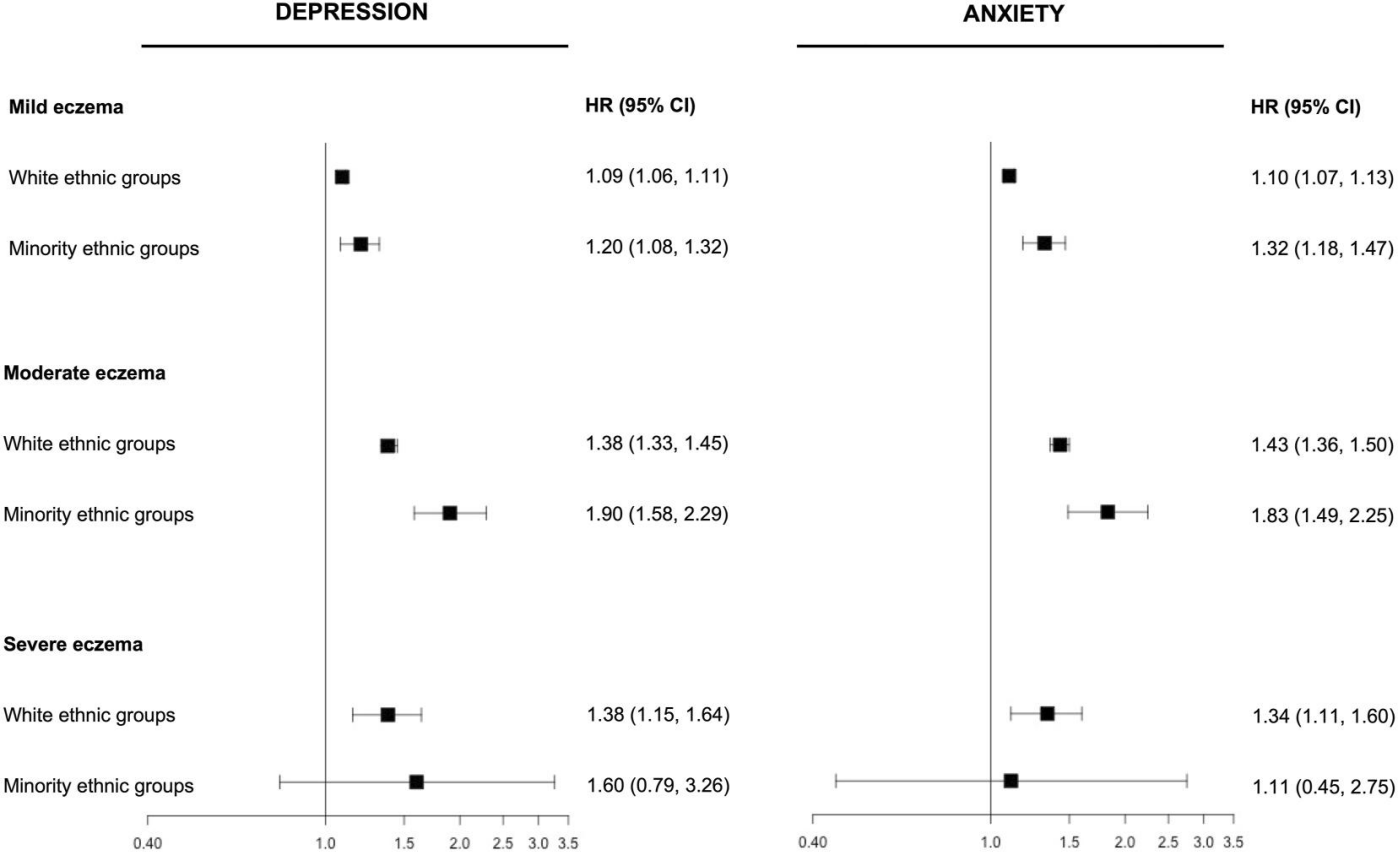
Associations (adjusted for potential confounders) between atopic eczema severity and incident depression and anxiety among adults in white and minority ethnic groups and adults in minority ethnic groups.

Individuals from white and minority ethnic groups spent similar proportions of total follow‐up at each level of eczema severity (Table S10). We saw evidence that at the same level of eczema severity, individuals from minority ethnic groups had higher hazards of depression and anxiety than adults from white ethnic groups (Table S11, Figure 2). This observation was particularly clear in associations between moderate eczema severity and depression (confounder adjusted HRs: minority ethnic groups: HR = 1.90, 95% CI = 1.58,2.29; white ethnic groups: HR = 1.38, 95% CI = 1.33,1.45) and anxiety (confounder adjusted HRs: minority ethnic groups: HR = 1.83, 95% CI = 1.49,2.25; white ethnic groups: HR = 1.43, 95% CI = 1.36,1.50), which were higher in minority ethnic groups compared to white ethnic groups.

## 4 DISCUSSION

We found that associations between eczema and depression or anxiety were more pronounced in adults from minority ethnic groups than in adults from white ethnic groups (after implicitly) adjusting for matching variables and adjusting for potential confounders (calendar period, deprivation). Estimated hazard ratios remained higher in adults from minority ethnic groups even after further adjusting for potential mediators (CCI, comorbid asthma, harmful alcohol use, smoking status, BMI, sleep problems, high‐dose glucocorticoid use). There were no clear differences in eczema severity between individuals from white and minority ethnic groups. At the same level of eczema severity, individuals from minority ethnic groups had higher risks of depression and anxiety than adults from white ethnic groups.

### 4.1 Strengths and limitations

To our knowledge, this is the first study to investigate whether associations between eczema and incident depression or anxiety differ between adults from white and minority ethnic groups. We identified adults with eczema in primary care and their ethnic groups using validated definitions.^33^, ^37^ We excluded individuals with relevant mental health conditions prior to cohort entry to ensure clarity of the temporal association between eczema and depression and anxiety across ethnic groups. CPRD GOLD is broadly representative of the UK population,^31^ suggesting that our results are broadly generalisable to the UK population with eczema.

However, our study has limitations. We did not match adults with eczema to those without eczema on ethnicity to prevent introducing selection bias; however, in an appropriate population with more complete ethnicity recording, this would allow a more accurate estimate of the interaction between eczema and ethnicity. The definition used to identify eczema (a combination of at least one diagnostic code, and at least two records of skin disease therapies) may introduce selection bias as it excludes untreated adults who may have milder disease. Further, our eczema definition required the presence of a diagnostic code; however, not all individuals with eczema will consult their general practitioner, meaning that they could be incorrectly classified as not having eczema. Misclassification of eczema in this study is likely to be related to the ascertainment of depression and anxiety, as individuals who do not consult their general practitioners for eczema may also not consult for depression or anxiety.

Both the misclassification of eczema, and ascertainment of depression and anxiety are also likely to be different in individuals from white and minority ethnic groups. Eczema appears different in skin of colour,^24^ and this visual difference can lead to misdiagnosis and subsequent misclassification of eczema exposure in individuals with skin of colour from minority ethnic groups. Additionally, evidence suggests that individuals from minority ethnic groups are more likely to have their depression and anxiety missed in primary care,^43^, ^44^, ^45^ either because health seeking behaviour is affected (by stigma or beliefs associated with mental health conditions),^46^ due to greater uncertainty by clinicians in diagnosing depression and anxiety,^47^ or because of a focus on physical health conditions despite the presence of mental health symptoms.^47^ In the context of this study, misclassification of eczema exposure and reduced ascertainment of depression and anxiety in individuals from minority ethnic groups may have biased our estimates of associations between eczema and depression or anxiety in individuals from minority ethnic groups towards the null, and true estimates of associations may be higher.

It could be argued that ascertainment of depression and anxiety may be more likely in adults with diagnosed eczema due to increased contact with primary care because of their skin disease, and this may lead to potential overestimates of associations between eczema and depression or anxiety in white and minority ethnic groups. However, our sensitivity analyses restricting study participation to adults with at least one primary care consultation in the year before cohort entry produced similar results to the main analysis, suggesting a minimal effect to our results. Although we excluded individuals with relevant mental health conditions prior to cohort entry, we cannot exclude that some individuals may have had depression or anxiety prior to their current GP registration. However, this is unlikely to have affected many individuals in our study as those with existing depression and anxiety diagnoses would be recorded as such during new patient consultations. Additionally, incorrectly classifying individuals as not having depression or anxiety at cohort entry is unlikely to be different in individuals with or without eczema, or individuals from white and minority ethnic groups, and will therefore have minimal impact on our results.

Our eczema severity definition may have misclassified adults with severe disease as having milder disease if they did not receive treatment. Additionally, individuals with more severe disease from minority ethnic groups may have been misclassified as having less severe disease as redness, a key identifier of disease severity, is difficult to see in people with darker skin.^24^ Misclassification of eczema severity is likely to lead to underestimates of associations between eczema severity and depression or anxiety in white and minority ethnic groups. Misclassification of eczema severity may also have reduced the number of individuals classified as having severe disease, underpowering our analyses and affecting the precision of estimates of associations between severe eczema and depression or anxiety in minority ethnic groups.

We excluded individuals with missing ethnicity data from both our depression and anxiety cohorts; however, individuals with missing ethnicity were more likely to be younger, male, and have missing BMI and smoking status. However, it is unlikely that excluding these individuals affected our results as multiple imputation of missing ethnicity data (Appendix S5) produced broadly similar results to those in the main analysis.

We pooled individuals from Black, South Asian, Mixed and Other ethnic groups into a minority ethnic group because of limited power. Pooling individuals meant that we were unable to investigate associations between eczema and incident depression and anxiety in the specific ethnic groups. Grouping individuals from several ethnic groups into one group and using an umbrella term such as ‘minority ethnic’ implies that they reflect a singular homogenous ethnic identity even though there is significant diversity between them. The white ethnic group in this study includes individuals from white minority groups (Gypsy, Roma, and Irish Traveller groups) who may also experience inequalities that may lead to differences in associations between eczema and depression or anxiety compared to individuals of White British ethnicity. However, investigating the inequalities experienced by white minority groups was limited by low statistical power.

We considered asthma as a potential mediator of associations between eczema and depression or anxiety, rather than a confounder as evidence suggests eczema and asthma occur together,^48^, ^49^ and reports of increased prevalence and incidence of depression and anxiety in people with asthma compared to the general population.^50^ However, even after adjusting our effect estimates for potential mediators, including asthma, the associations between eczema and depression or anxiety were attenuated but remained higher among adults from minority ethnic groups.

Our mediator‐adjusted estimates of associations between eczema and depression or anxiety may include residual effects of incompletely captured and uncaptured mediators. For example, sleep problems are likely to be imperfectly captured in routinely collected primary care data, because individuals do not always consult their general practitioner for sleep problems. Additionally, cultural beliefs about skin disease, discrimination and inequalities experienced by individuals from minority ethnic groups are important factors that could potentially mediate associations; however, they cannot be captured using CPRD data. Further, we captured some potential mediators on or before index date, and consequently some mediators may have been captured before eczema diagnosis,^51^ meaning they cannot be on the causal pathway after exposure as our analysis strategy assumes. However, given that eczema frequently starts in childhood and most individuals with eczema did not enter the cohorts on the date of the first eczema diagnosis, and it is unlikely that the measurement of most included mediators (e.g., BMI, smoking status) changes over time, the timing of capture of our mediators may have a limited effect on our mediator‐adjusted estimates.

### 4.2 Comparisons to existing literature

There is limited existing evidence on the role of ethnicity in associations between eczema and depression or anxiety. Our study addresses the limitations of previous research by specifically estimating HRs for associations between eczema and depression or anxiety in white and minority ethnic groups. Our finding that adults from minority ethnic groups are at an increased risk of depression or anxiety compared to those from white ethnic groups is consistent with studies conducted in the general population where rates of depression, anxiety, and other mental health conditions are much higher in minority ethnic communities.^25^, ^26^, ^52^, ^53^

One possible explanation for differences in associations with depression or anxiety may be due to potential differences in how eczema is diagnosed and managed in different ethnic groups. Eczema appears different in skin of colour, which may lead to delayed diagnosis, underestimation of disease severity, or even misdiagnosis among people with darker skin from minority ethnic groups.^24^ Our eczema and severity definitions are also based on skin disease therapies, and differences in eczema diagnoses in skin of colour may subsequently lead to undertreatment or delayed treatment and an increased risk of mental health conditions. We did not observe a clear difference in eczema severity between adults of white and minority ethnic groups, a finding that is inconsistent with studies reporting greater eczema severity in individuals from minority ethnic groups.^54^, ^55^ The difference in findings may be explained by differences in the eczema severity definitions used across studies. Our severity definition was based on treatment with skin disease therapies, while in other studies,^54^, ^55^ severity was assessed through surveys or clinical scoring tools. Additionally, the study populations of previous studies included only children and adolescents, potentially indicating that ethnic differences in eczema severity may be different to adulthood.

### 4.3 Implications for research and clinical practice

Our findings suggest that individuals with eczema from minority ethnic groups are at higher risk of depression and anxiety and highlight the importance of monitoring mental health in this vulnerable population. Monitoring mental health and wellbeing in individuals from minority ethnic groups with eczema is vital, since individuals from these communities in the general population are already at comparatively higher risk of depression and anxiety than people from white communities,^25^, ^26^, ^52^, ^53^ and an eczema diagnosis may further increase the risk.

Unrecognised depression or anxiety in people with eczema from minority ethnic groups may reduce skin disease treatment adherence,^56^ therefore reducing treatment benefits, potentially worsening skin disease, and subsequently contributing to worsening mental health. Introducing mental health promotion strategies as targeted mental health screening in primary care of adults with eczema from minority ethnic groups may avoid the development of depression and anxiety, or in individuals already affected, may allow early detection and intervention. Ensuring mental health promotion and assessment is ethnically and culturally appropriate (i.e., sensitive to the beliefs and the expression of symptoms in individuals from minority ethnic groups) may be one of several equitable solutions that lead to improved recognition of depression and anxiety among individuals from minority ethnic groups. Equitable support may also ensure that the risk of mental health conditions among individuals with eczema is equal among individuals from different ethnic groups. These mental health promotion and prevention strategies may be best delivered through individualised care that recognizes the varied issues that individuals from different cultural and ethnic backgrounds may face. Several systematic reviews have reported that the use of culturally sensitive mental health assessments and interventions effectively improve health outcomes (e.g., through community outreach and involvement interventions to ensure individuals are aware of available mental health services, reducing stigma associated with use of mental health services, and improving overall well‐being).^57^, ^58^ Improving the recognition of eczema among individuals from minority ethnic groups may also contribute to reducing depression and anxiety risk in this population. As eczema appears different in skin of colour,^24^ and the visual difference can lead to misdiagnosis, training clinicians to identify eczema in skin of colour may lead to earlier diagnosis and improved management of eczema, and consequently better mental health outcomes.

Future work could investigate differential health seeking behaviours and dermatological referrals for those in white and minority ethnic groups, and investigate whether these may explain the increased risk of depression and anxiety among individuals from minority ethnic groups.

## 5 CONCLUSIONS

Adults with eczema from minority ethnic groups appear to be at increased risk of depression or anxiety compared with their white counterparts. Improved training for clinicians in identifying eczema in individuals with skin of colour may reduce misdiagnosis among individuals with darker skin types, leading to better management and improved mental health outcomes. Mental health promotion and prevention strategies that are culturally adapted and targeted to individual patients may be one of several equitable solutions to reduce the burden of mental health conditions in individuals with eczema from minority ethnic groups, aiming to minimise inequalities in the risk of mental health conditions among individuals with eczema among individuals from different ethnic groups.

## AUTHOR CONTRIBUTIONS

Elizabeth I. Adesanya, Alasdair Henderson, Sinéad M. Langan and Kathryn E. Mansfield had the original idea for the study. Elizabeth I. Adesanya carried out the statistical analysis and wrote the first draft. All authors contributed to further drafts and approved the final manuscript.

## CONFLICT OF INTEREST STATEMENT

Joseph F. Hayes has received consultancy fees from Wellcome Trust and Juli Health. Rohini Mathur and Kathryn E. Mansfield have received consultancy fees from AMGEN.

## Supporting information

Supporting Information S1

## Data Availability

Data may be obtained from a third‐ party and are not publicly available. Data from this study were obtained from the Clinical Practice Research Datalink (CPRD) and cannot be shared directly by researchers. Data are available directly from CPRD subject to independent approval.
