# Supplementary material for: Ethnic differences in depression and anxiety among adults with atopic eczema: Population‐based matched cohort studies within UK primary care
Source: Clin Transl Allergy. 2024 Mar 25;14(3):e12348. doi: 10.1002/clt2.12348 (PMC10962487; doi:10.1002/clt2.12348)
Supplement: Supplementary file 1 — Supporting Information S1 [file CLT2-14-e12348-s001.docx]

**Supplementary material - Ethnic differences in depression and anxiety among adults with atopic eczema: population-based matched cohort studies**

**Authors:** Elizabeth I Adesanya, Alasdair Henderson, Joseph Hayes, Rohini Mathur, Caroline Morton, Sinéad Langan, Kathryn Mansfield

**Appendix S1:** Explanation of matching without replacement in calendar date order

**Appendix S2:** Directed acyclic graph (DAG) of the implicitly assumed causal relationships between atopic eczema and depression/anxiety, and explanation of the variables included in the DAG

**Appendix S3:** Variable definitions

**Appendix S4:** Testing the proportional hazards assumption using Schoenfeld residual plots

**Appendix S5:** Multiple imputation of missing ethnicity data

**Appendix S6:** Secondary analyses

**References**

**Table S1:** Description of sensitivity analyses, and HR (95% CI) of sensitivity analyses in depression cohort

**Table S2:** Description of sensitivity analyses, and HR (95% CI) of sensitivity analyses in anxiety cohort

**Table S3:** Characteristics of main analysis cohort, HES-enriched sensitivity cohort, and multiple imputation of missing ethnicity cohort used to investigate associations between atopic eczema and depression in white and minority ethnic groups

**Table S4:** Characteristics of main analysis cohort, HES-enriched sensitivity cohort, and multiple imputation of missing ethnicity cohort used to investigate associations between atopic eczema and anxiety in white and minority ethnic groups

**Table S5:** Comparison of baseline characteristics of individuals with recorded and without recorded ethnicity in depression and anxiety cohorts

**Table S6:** Person-time under follow-up in depression and anxiety cohorts broken down by individual-level characteristics and atopic eczema exposure status

**Table S7:** Characteristics of the depression cohort at cohort entry, for: the overall cohort, individuals included in the model additionally adjusting for potential confounders (i.e., individuals with no missing Carstairs deprivation data), individuals with missing Carstairs data, individuals included in the model additionally adjusting for potential mediators (i.e., individuals with no missing BMI or smoking status data), and for individuals with missing BMI or smoking status

**Table S8:** Characteristics of the anxiety cohort at cohort entry, for: the overall cohort, individuals included in the model additionally adjusting for potential confounders (i.e., individuals with no missing Carstairs deprivation data), individuals with missing Carstairs data, individuals included in the model additionally adjusting for potential mediators (i.e., individuals with no missing BMI or smoking status data), and for individuals with missing BMI or smoking status

**Table S9:** HRs (95% CI) for the association between atopic eczema and depression or anxiety. Fitted to adults with complete data for all variables included in each model and from valid matched sets

**Table S10:** Proportions of total follow up each ethnic group (white or minority ethnic) spends at each level of atopic eczema (mild, moderate, or severe) severity during follow up. Data are n (%)

**Table S11:** HRs (95% CI) for the association between atopic eczema severity and depression or anxiety in white and minority ethnic groups.

**Appendix S1 – Explanation of matching without replacement in calendar date order**

In this study, we matched adults with atopic eczema (on age, sex, and practice) with up to five adults without atopic eczema without replacement and in calendar date order. We matched one individual with atopic eczema with up to five without to increase the precision of estimates of effect. Beyond five matched comparators, there is little gain in efficiency.

Matching without replacement means that each of the up to five adults without atopic eczema were matched to only one adult with atopic eczema. This contrasts to matching with replacement, where the matched comparators (i.e., those without atopic eczema) could be matched to multiple individuals with atopic eczema. Matching with replacement allows sample size to be maximised as fewer exposed people (those with atopic eczema) are excluded because of lack of comparators and a high matching ratio can be ensured. However, as we had a large pool of eligible comparators (and were consequently not limited by sample size considerations, and able to preserve our matching ratio), we were not limited to matching with replacement. We chose to match without replacement as matching with replacement can result in substantial reuse of comparators meaning that standard errors become too optimistic and confidence intervals artificially narrow.

By ‘matching in calendar date order’ we mean that unexposed individuals (those without atopic eczema) in the matched cohort were assigned first to exposed individuals with the earliest cohort entry. Matching in calendar date order avoids some time-related bias.

**
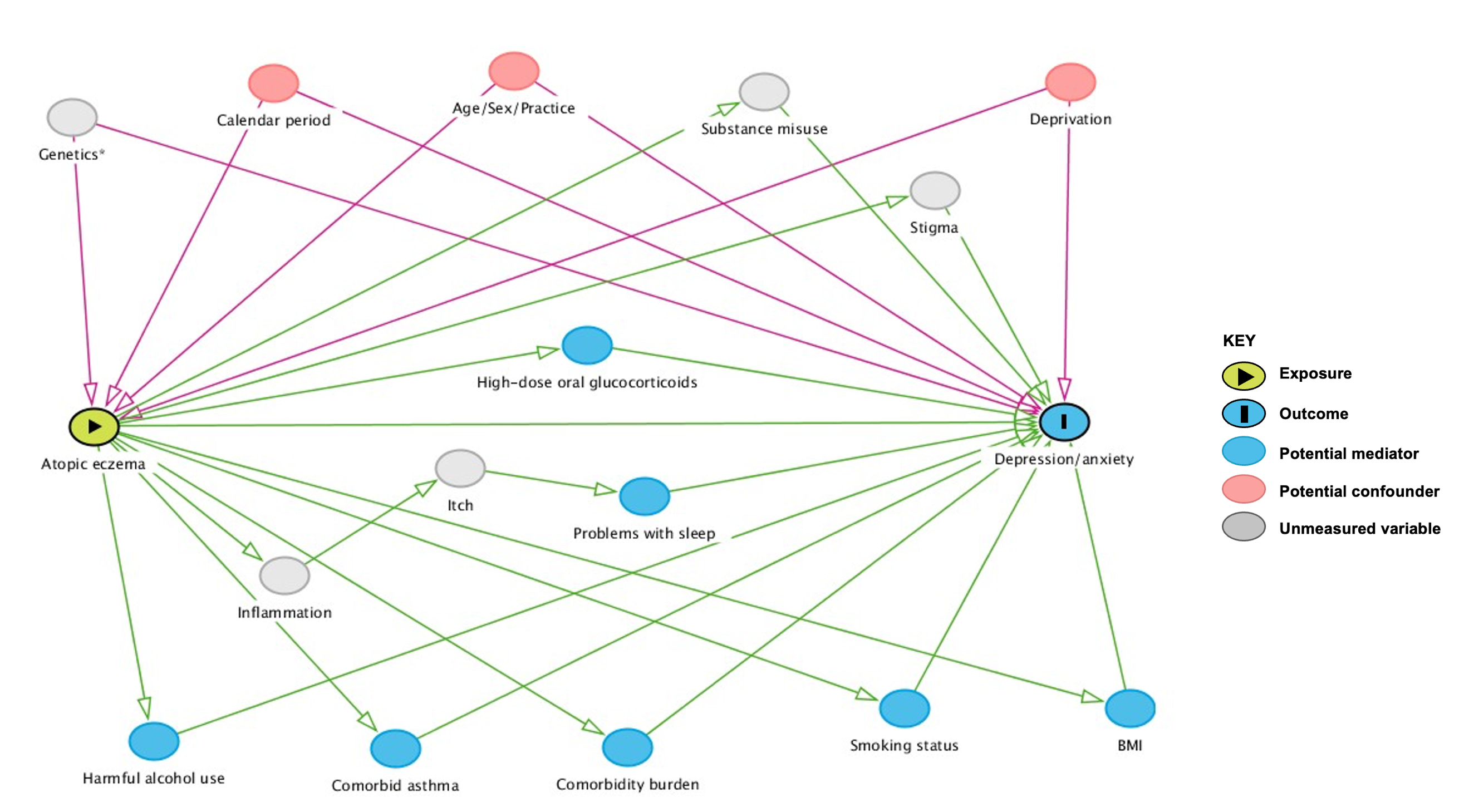
Appendix S2 – Directed acyclic graph (DAG) of the implicitly assumed causal relationships between atopic eczema and depression or anxiety, and explanation of the variables included in the DAG**

Explanation of variables included in directed acyclic graphs of the relationships between atopic eczema and depression or anxiety

| **Variables** | **Type** | **Measured/Unmeasured** | **Justification** |
| --- | --- | --- | --- |
| Age/Sex/Practice | Potential confounder (matching variables) | Measured | Atopic eczema can occur at any age, however, it is more prevalent in younger people (the usual age of onset is in early childhood), ^1,2^ and women compared to men.^3,4^ The prevalence of depression peaks in older adulthood (individuals aged between 55-74 years), but it can also occur at a lower level than adults in children and adolescents below the age of 15.^5^ On the other hand, prevalence rates of anxiety do not differ considerably between age groups, however, prevalence tends to decrease among older age groups.^5^ Depression and anxiety are more common among women than men.^5–7^ As these variables were not on the causal pathway in the association between atopic eczema and depression or anxiety, they were considered as potential confounders and used to match individuals in the study. We also matched on practice to control for clinical and administrative practices that may differ between general practitioners. |
| BMI | Potential mediator | Measured | Evidence suggests that a BMI is associated with atopic eczema. A population-based study in the UK using primary care data found that people with atopic eczema had 8% higher odds of being overweight or obese compared to those without atopic eczema.^8^ Higher BMI is also strongly associated with increased risk of depression and anxiety,^9,10^ with evidence from Mendelian randomisation studies suggesting a causal mechanism.^11^ Due to this evidence, we considered BMI a potential mediator of associations between atopic eczema and depression or anxiety. |
| Calendar period | Potential confounder | Measured | The relationship between atopic eczema and depression or anxiety may be influenced by various calendar-based factors such as changes in clinical and administrative practices in atopic eczema, depression, and anxiety over time. For example, after the introduction of performance indicators for depression in the Quality and Outcomes Framework (QOF) in 2006, GP recording of depression or anxiety have been altered, and the use of symptom codes for both depression and anxiety have increased.^12^ Adjusting for calendar period as a confounder in our analysis will allow us to remove any possible calendar variation and effectively assess the true association between atopic eczema and depression or anxiety. |
| Comorbid asthma | Potential mediator | Measured | Atopic eczema has been found to be associated with, or predispose affected individuals to, numerous atopic comorbidities including asthma.^13–16^ Asthma is also known to be associated with anxiety and depression, with the literature reporting a higher prevalence and incidence of depression and anxiety in people with asthma compared to the general population.^17^ Due to this evidence, we considered comorbid asthma a potential mediator of associations between atopic eczema and depression or anxiety. |
| Comorbidity burden | Potential mediator | Measured | Atopic eczema has been found to be associated with several atopic comorbidities (e.g., asthma and allergic rhinitis),^13–16^ cardiovascular outcomes (e.g., hypertension, coronary heart disease, heart failure) which may partly relate to lifestyle habits (i.e., sedentary lifestyle, diet, harmful alcohol consumption),^8,13,18–21^ infections,^22,23^ and fractures.^24^  The relationships between comorbidity burden and depression or anxiety are often complex and bidirectional. Long-term physical health problems such as cancer, diabetes, cardiovascular disease, and other chronic conditions can be distressing and lead to depression or other psychological problems in affected individuals. For example, anxiety is common in people with chronic respiratory diseases, while depression is more common in people with diabetes.^25^ However, there is also evidence that mental health conditions can increase the risk of chronic conditions. Evidence from systematic reviews and meta-analyses suggest that depression and anxiety disorders are independent risk factors for diabetes and coronary heart disease.^25^  What we can see from the evidence is that long term physical health conditions and depression or anxiety often co-occur, and the direction of the relationship between both conditions is complex and difficult to understand. In the context of this study, we considered comorbidity burden as a potential mediator of associations between atopic eczema and depression or anxiety, and excluded comorbidities recorded after the mental health outcome had occurred from analyses. |
| Deprivation | Potential confounder | Measured | Multiple studies have found that a higher socioeconomic status (and thus lower levels of deprivation) in children is associated with an increased prevalence of atopic eczema,^3,26^ while in adulthood, prevalence was either unaffected by socioeconomic status or higher in individuals with lower socioeconomic status.^3,4^ There is also strong evidence that people of lower socioeconomic status (and high levels of deprivation) are more likely to develop and experience mental health problems such as depression and anxiety.^7,27^ Therefore, in this study, we considered deprivation a potential confounder of associations between atopic eczema and depression or anxiety. |
| Genetics (atopic eczema and depression only) | Potential confounder | Unmeasured | Limited studies have suggested a shared genetic link between atopic eczema and depression.^28^ This relationship is not on the causal pathway, suggesting that genetics are a potential confounder of the association between atopic eczema and depression, or that a shared genetic aetiology is the reason for any observed association. However, genetic information is not routinely recorded in primary care records, therefore this variable is unmeasured. To my knowledge, a genetic association between atopic eczema and anxiety has not been established. |
| Harmful alcohol use | Potential mediator | Measured | Evidence shows that individuals with atopic eczema consume more alcohol than the general population and subsequently have a higher prevalence of alcohol use disorders compared to populations without skin disease.^21,29^ Harmful alcohol use and alcohol dependence have been regularly associated with symptoms of depression and anxiety.^30,31^ Due to this evidence, we considered harmful alcohol use a potential mediator of associations between atopic eczema and depression or anxiety. |
| High-dose oral glucocorticoids | Potential mediator | Measured | High dose oral glucocorticoids (≥20mg/day prednisolone equivalent dose) such as prednisolone, dexamethasone, and hydrocortisone may be used for short-term use in individuals where topical treatments or phototherapy have failed, or in people with severe atopic eczema flares.^22,32^ During therapy, glucocorticoids can induce psychiatric side effects including symptoms of depression, anxiety, mania, and psychosis, however, their effect is temporary.^33^ Due to this evidence, we considered high-dose oral glucocorticoid use a potential mediator of associations between atopic eczema and depression or anxiety. |
| Inflammation | Potential mediator | Unmeasured | Atopic eczema is a chronic inflammatory skin disease characterised by elevated levels of circulating pro-inflammatory cytokines.^32^ Evidence also suggests that as atopic eczema severity increases, so do the levels of inflammatory markers.^34^ Both depression and anxiety have been associated with increased inflammatory response of the immune system,^35^ and clinical trials of biologics that target inflammatory cytokines in those with atopic eczema have found that these drugs may also be associated with a reduction in symptoms of depression and anxiety.^36^ Inflammation can therefore be considered a potential confounder of the relationship between atopic and depression or anxiety. However, inflammation is not recorded in CPRD GOLD, so it is an unmeasured variable. However, in people with atopic eczema, inflammation can lead to itch and cause sleep problems in those affected |
| Itch and problems with sleep | Potential mediator | Measured | In people with atopic eczema, sleep disturbances are a common and well-recognised consequence of severe itch. Chronic and intense itching is a major clinical manifestation of atopic eczema that continues throughout the day and gets worse at night, leading to sleep disturbance or deprivation.^2^ Studies have found that people with atopic eczema are more likely to report fatigue, regular insomnia, shorter sleep duration, and daytime sleepiness when compared to the general population.^37^ Longitudinal evidence has identified sleep disturbances as risk factors for the development of depression and anxiety.^38^ However, itch is not recorded in CPRD GOLD, so it is an unmeasured variable. On the other hand, problems with sleep can be captured in CPRD GOLD, although they are likely to be an underestimate of sleep problems. Due to this evidence, we considered problems with sleep a potential mediator of associations between atopic eczema and depression or anxiety. |
| Smoking status | Potential mediator | Measured | Evidence suggests that atopic eczema is associated with smoking. Adults with atopic eczema are 28% more likely to be current smokers compared to the general population.^21^ Smoking rates are also high in individuals with depression and anxiety, with some longitudinal evidence suggesting that smoking leads to later development of depression or anxiety.^39^ Specifically in individuals with atopic eczema, a relationship has been demonstrated between smoking status and an increased risk of depression.^40^ Due to this evidence, we considered smoking status a potential mediator of associations between atopic eczema and depression or anxiety. |
| Stigma | Potential mediator | Unmeasured | In a survey of adults with atopic eczema, respondents indicated perceiving stigma in social events, employment, romantic relationships and self-image.^41^ Stigma can exacerbate negative emotions and impact the self-esteem of those affected, consequently leading to mental health outcomes.^41^ Stigma has been found to be a predictor for depressive symptoms and psychological factors in people with atopic eczema.^41,42^ Stigma can therefore be considered a potential mediator of associations between atopic eczema and depression or anxiety, however, it is not recorded in CPRD GOLD; therefore, it is considered an unmeasured variable. |
| Substance misuse | Potential mediator | Unmeasured | Substance misuse can be defined as the use of illegal psychoactive drugs, or the use of prescription or over-the-counter medication for purposes other than those they are meant for.^43^ Evidence suggests that people with atopic eczema are more likely than people in the general population to misuse substances such as cannabis,^44^ potentially due to the powerful anti-itch effect of cannabinoids that could potentially reduce the symptoms and appearance of atopic eczema.^44^ Heavy and regular use of cannabis is also associated with an increased risk of depressive symptoms.^45^ Substance misuse can therefore be considered a potential mediator of associations between atopic eczema and depression or anxiety.  However, studies have reported that substance misuse is recorded in primary care at lower rates compared to national surveys.^46^ There are also stigmas associated with substance misuse,^43^ so affected individuals may not feel comfortable discussing substance use with their GPs. Substance misuse is therefore incompletely captured in CPRD GOLD and has been considered an unmeasured variable. |

BMI – Body mass index; CPRD – Clinical Practice Research Datalink

**Appendix S3 – Variable definitions**

**Atopic eczema**

We identified atopic eczema using a previously validated definition based on a record of at least one diagnostic code recorded in primary care and at least two records of eczema therapy recorded (in primary care using Read codes or prescription data) on separate days.^47^ Eczema therapy included: (1) records of phototherapy identified using Read codes in primary care and (2) primary care prescription records for topical emollients, corticosteroids or calcineurin inhibitors, or oral glucocorticoids, azathioprine, methotrexate, ciclosporin or mycophenolate.

**Depression and anxiety**

We identified depression and anxiety based on the earliest record of a diagnostic or symptom Read code recorded in primary care. We considered broader definitions of depression and anxiety in sensitivity analyses.

**Ethnicity**

We identified ethnicity using a previously validated algorithm that identifies ethnicity using primary care electronic health records.^48^ The algorithm classifies ethnicity into five categories – White, South Asian, Black, Other or Mixed – and is suggested for use with the CPRD dataset from 2006 onwards to maximise completeness and comparability of data. We pooled individuals from Black, South Asian, Mixed and Other ethnic groups into a ‘minority ethnic’ group. We used this grouping to investigate whether associations between atopic eczema and depression/anxiety differed between individuals from white and minority ethnic groups.

**General practice**

We matched on general practice as an indirect method to capture and adjust for general practice location (i.e., rural, or urban locations) and socioeconomic deprivation. Matching individuals on general practice also allowed us to account for differences in coding practices between GPs.

**Calendar period**

Calendar period was categorised as 2006-2010, 2011-2015, and 2016-2020 to account for changes in clinical, diagnostic, and administrative practices over the study period that may have influenced the measurement of exposure, outcomes, and other covariates.

**Comorbidity burden**

We used the Charlson Comorbidity Index (CCI) as a summary measure to capture the burden of comorbidities recorded on or before index date. The CCI is a method of categorising comorbidities of individuals that assigns weights to each of the 17 conditions included in the index, and then sums the weights of those conditions present in the individual.^49–51^ Each condition in the CCI is weighted from one to six, with a weight of six representing the most severe morbidity.^49,51^ The sum of the weights in each individual results in a single comorbidity summary score. We categorised CCI scores into 3 groups: low (0 points), intermediate (1-2 points), and high (≥3 points).

**Comorbid asthma**

We identified adults with comorbid asthma based on morbidity coding in primary care. Individuals were regarded as having asthma from the earliest record of a relevant diagnostic code.

**Deprivation**

We used the Carstairs Index (CI) as a proxy for socioeconomic deprivation. The Carstairs Index was measured using quintiles of the individual-level Carstairs scores from 2011 census data linked via the individual postal code. Patient level Carstairs data was only available for people in English practices that consented to participate in the linkage scheme. When individual-level data was unavailable, we used practice-level data.

**Harmful alcohol use**

We defined harmful alcohol use based on primary care morbidity codes suggesting harmful or heavy alcohol use (including alcohol dependency codes and codes related to physical/psychological harm related to alcohol use) or a prescription for drugs used to maintain abstinence (acamprosate, disulfiram, or nalmefene). Individuals were defined as harmful alcohol users on the date of the first record of a relevant morbidity code or prescription.

**High-dose oral glucocorticoid use**

We identified prescriptions for oral glucocorticoids (prednisolone, betamethasone, deflazacort, dexamethasone, hydrocortisone, methylprednisolone, prednisone, triamcinolone, and cortisone) and converted the prescribed daily dose to the prednisolone-equivalent dose (PED). High-dose oral glucocorticoid use was defined as a dose of 20 mg/day or higher PED. We captured high-dose oral glucocorticoid use as a binary time-updated variable with status changing for a short period (90 days) on the date of the first record of a prescription for a dose of 20mg/day or more PED.

**Sleep problems**

We identified sleep problems based on primary care morbidity codes suggesting sleep problems and prescriptions for drugs used to manage sleep problems. In our main analysis, this included diagnostic Read codes (for insomnia, sleep disorders, poor sleep pattern, or other sleep disturbances) and prescriptions for drugs that are only used to treat sleep problems (zaleplon, zolpidem tartrate, zopiclone, hydroxyzine hydrochloride and promethazine hydrochloride when taken at night). In our sensitivity analysis, we used alternative code lists including prescriptions for drugs that may be prescribed for sleep problems but can also be prescribed for other conditions (melatonin and benzodiazepines).

**Smoking status and body mass index (BMI)**

We defined BMI and smoking status using an algorithm using primary care records to identify the status recorded closest to the cohort entry date. The algorithm regarded records identified within -1 year to +1 month of the index date as the best, +1 month to +1 year from the index date as second best, the nearest before -1 year from the index date as the third best, and the nearest after +1 year from the index date as the worst. We did not include smoking status or BMI recorded after the outcome had occurred. Smoking status was classified as: (1) current/ex-smoker; or (2) non-smoker. BMI was classified according to the World Health Organisation categories: underweight (<18.5kg/m2); normal weight (18.5-24.9 kg/m2); pre-obesity (25.0-29.9 kg/m2); obese (≥30.0 kg/m2). Smoking status or BMI recorded after depression or anxiety diagnoses were not used.

**Appendix S4 – Testing proportional hazards assumption using Schoenfeld residual plots**

We tested the proportional hazards assumption of our confounder-adjusted Cox regression models (adjusted for deprivation and calendar time) using Schoenfeld residual plots. In the confounder-adjusted models for white and minority ethnic groups in the depression and anxiety cohorts, there was no evidence that the proportional hazards assumption was violated.

**Confounder-adjusted model in the depression cohort (white ethnic group): p = 0.6832**


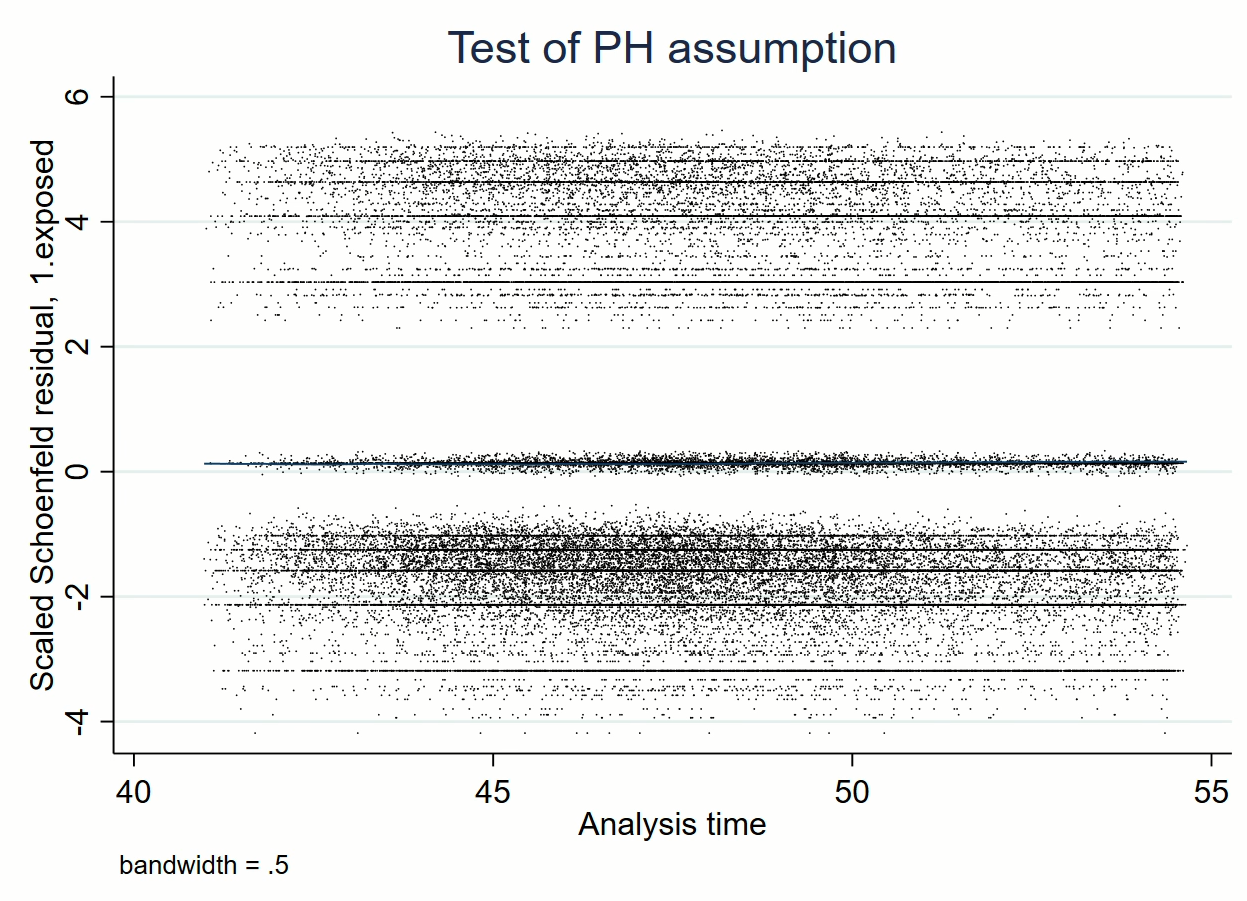


**Confounder-adjusted model in the depression cohort (minority ethnic group): p = 0.2689**


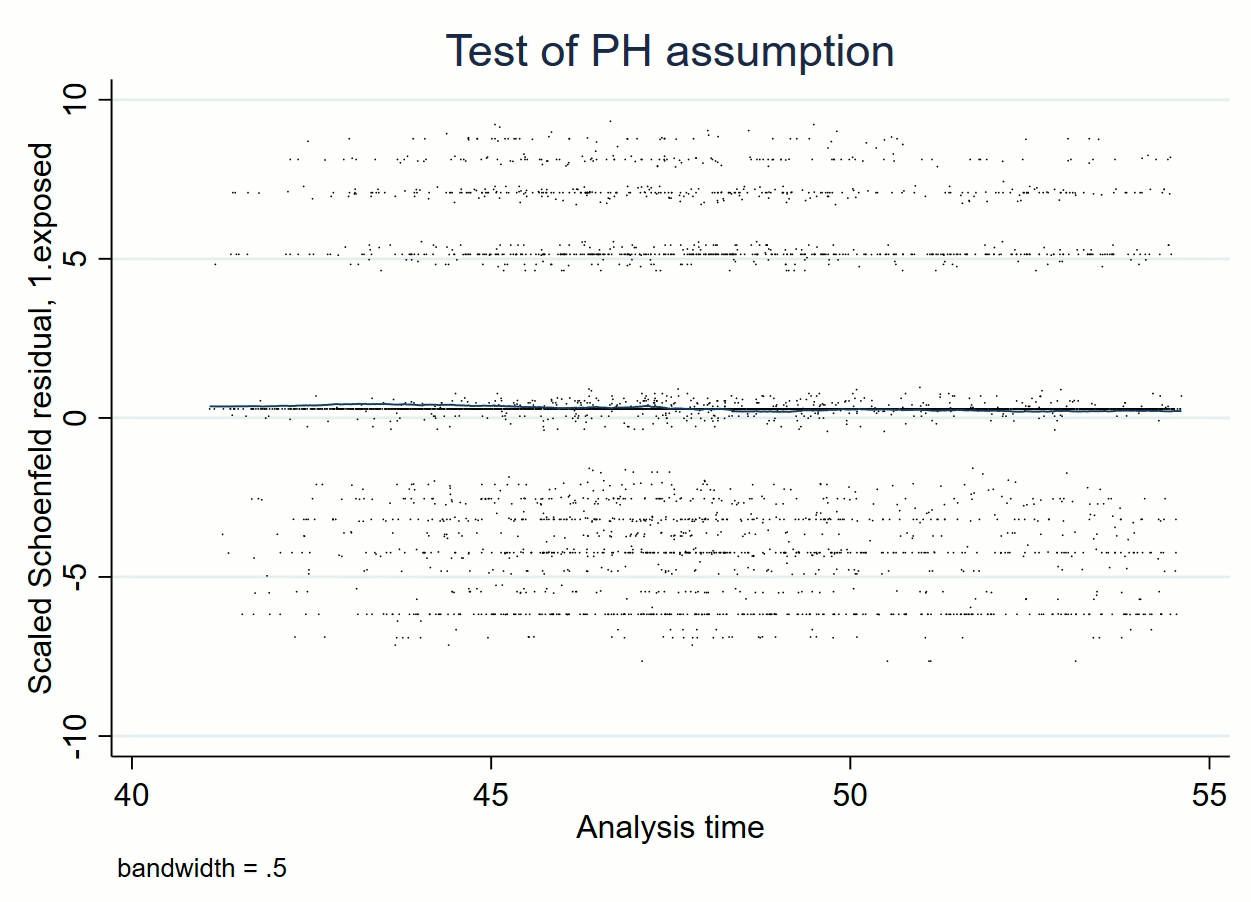


**
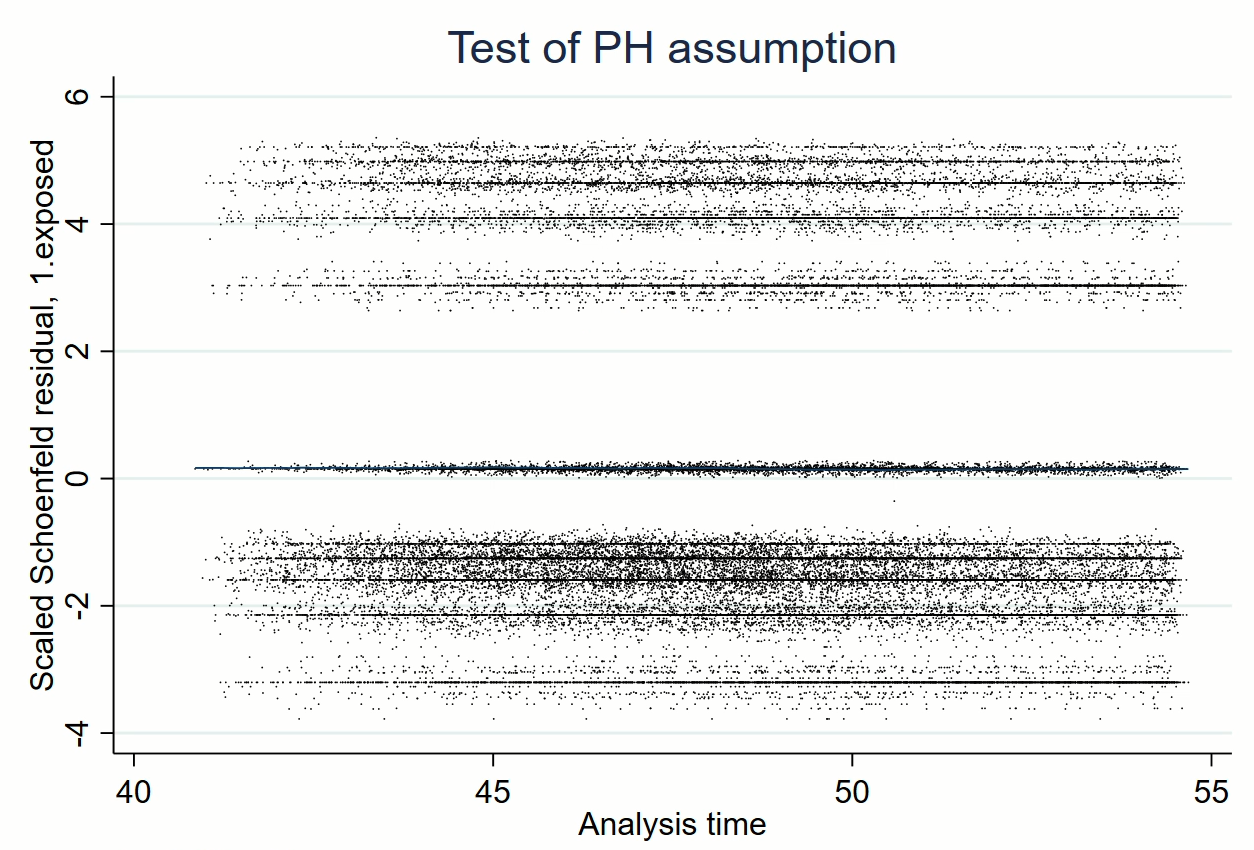
Confounder-adjusted model in the anxiety cohort (white ethnic group): p = 0.9953**

**Confounder-adjusted model in the anxiety cohort (minority ethnic group): p = 0.9381**

**
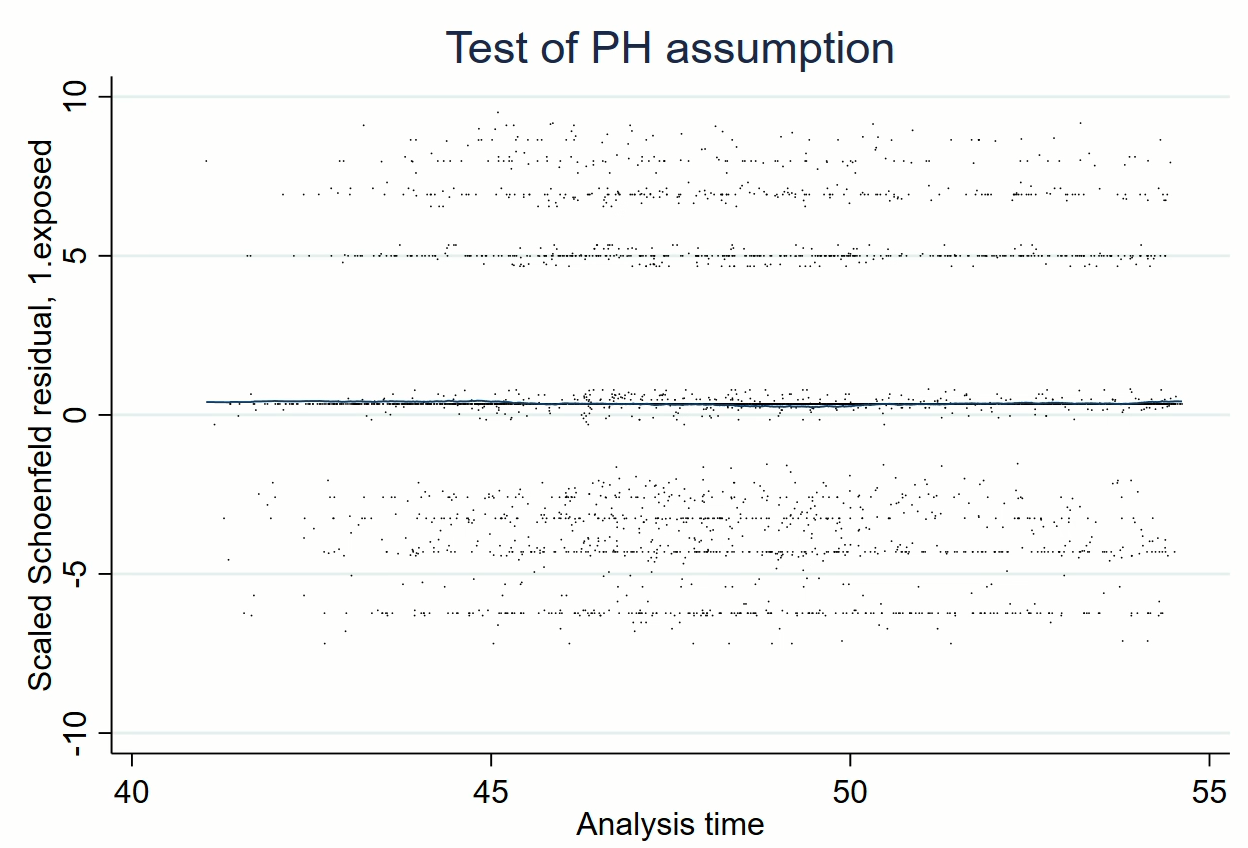
**

**Appendix S5 – Multiple imputation of missing ethnicity data**

**Imputation method**

We used a logistic regression imputation method to fill in missing values of the binary ethnicity variable (white ethnic group, minority ethnic groups). We created 10 imputations of the missing ethnicity variable, including potential confounders (deprivation and calendar period) in our imputation model. Although at least 20 imputations are recommended, we conducted 10 as more imputations above this were computationally intensive.

**Results of multiple imputation**

The table below shows the distribution of ethnicity across imputations in the depression and anxiety cohorts. Although numbers of individuals in each ethnic group varied across imputations, sample sizes vastly increased with on average over 430,000 adults with atopic eczema matched to over 1,700,000 adults without atopic eczema in the depression cohort, and over 480,000 adults with atopic eczema matched with over 2,000,000 without atopic eczema in the anxiety cohort.

| **Imputation** | **Depression cohort** | | **Anxiety cohort** | |
| --- | --- | --- | --- | --- |
|  | **Number** | **Events** | **Number** | **Events** |
| **IMPUTATION 1** |  |  |  |  |
| *White* |  |  |  |  |
| Without atopic eczema | 1,471,472 | 113,915 | 1,738,109 | 97,533 |
| With atopic eczema | 374,169 | 36,246 | 415,726 | 30,140 |
| *Minority ethnic* |  |  |  |  |
| Without atopic eczema | 243,800 | 9,764 | 273,242 | 7,932 |
| With atopic eczema | 62,384 | 3,449 | 66,475 | 2,633 |
| **IMPUTATION 2** |  |  |  |  |
| *White* |  |  |  |  |
| Without atopic eczema | 1,471,750 | 113,922 | 1,737,526 | 97,648 |
| With atopic eczema | 374,160 | 36,241 | 415,749 | 30,075 |
| *Minority ethnic* |  |  |  |  |
| Without atopic eczema | 243,430 | 9,757 | 273,799 | 7,817 |
| With atopic eczema | 62,180 | 3,454 | 66,535 | 2,698 |
| **IMPUTATION 3** |  |  |  |  |
| *White* |  |  |  |  |
| Without atopic eczema | 1,471,377 | 113,958 | 1,738,170 | 97,574 |
| With atopic eczema | 374,364 | 36,263 | 415,882 | 30,146 |
| *Minority ethnic* |  |  |  |  |
| Without atopic eczema | 243,835 | 9,721 | 273,174 | 7,891 |
| With atopic eczema | 62,182 | 3,432 | 66,187 | 2,627 |
| **IMPUTATION 4** |  |  |  |  |
| *White* |  |  |  |  |
| Without atopic eczema | 1,471,974 | 114,043 | 1,738,383 | 97,562 |
| With atopic eczema | 373,650 | 36,225 | 415,807 | 30,189 |
| *Minority ethnic* |  |  |  |  |
| Without atopic eczema | 243,322 | 9,636 | 272,899 | 7,903 |
| With atopic eczema | 62,817 | 3,470 | 66,308 | 2,584 |
| **IMPUTATION 5** |  |  |  |  |
| *White* |  |  |  |  |
| Without atopic eczema | 1,471,731 | 113,810 | 1,737,441 | 97,524 |
| With atopic eczema | 373,950 | 36,269 | 415,784 | 30,155 |
| *Minority ethnic* |  |  |  |  |
| Without atopic eczema | 243,416 | 9,869 | 273,812 | 7,941 |
| With atopic eczema | 62,514 | 3,426 | 66,330 | 2,618 |
| **IMPUTATION 6** |  |  |  |  |
| *White* |  |  |  |  |
| Without atopic eczema | 1,471,273 | 113,849 | 1,737,982 | 97,623 |
| With atopic eczema | 374,508 | 36,288 | 415,337 | 30,144 |
| *Minority ethnic* |  |  |  |  |
| Without atopic eczema | 243,825 | 9,830 | 273,296 | 7,842 |
| With atopic eczema | 61,900 | 3,407 | 66,883 | 2,629 |
| **IMPUTATION 7** |  |  |  |  |
| *White* |  |  |  |  |
| Without atopic eczema | 1,471,801 | 113,916 | 1,737,328 | 97,648 |
| With atopic eczema | 373,882 | 36,223 | 415,694 | 30,163 |
| *Minority ethnic* |  |  |  |  |
| Without atopic eczema | 243,321 | 9,763 | 273,903 | 7,817 |
| With atopic eczema | 62,729 | 3,472 | 66,372 | 2,610 |
| **IMPUTATION 8** |  |  |  |  |
| *White* |  |  |  |  |
| Without atopic eczema | 1,471,555 | 113,886 | 1,737,502 | 97,626 |
| With atopic eczema | 374,208 | 36,312 | 415,760 | 30,128 |
| *Minority ethnic* |  |  |  |  |
| Without atopic eczema | 243,596 | 9,793 | 273,787 | 7,839 |
| With atopic eczema | 62,125 | 3,383 | 66,397 | 2,645 |
| **IMPUTATION 9** |  |  |  |  |
| *White* |  |  |  |  |
| Without atopic eczema | 1,471,486 | 113,813 | 1,737,916 | 97,637 |
| With atopic eczema | 374,182 | 36,315 | 415,955 | 30,213 |
| *Minority ethnic* |  |  |  |  |
| Without atopic eczema | 243,794 | 9,866 | 273,243 | 7,828 |
| With atopic eczema | 62,309 | 3,380 | 66,097 | 2,560 |
| **IMPUTATION 10** |  |  |  |  |
| *White* |  |  |  |  |
| Without atopic eczema | 1,471,653 | 113,955 | 1,737,253 | 97,613 |
| With atopic eczema | 374,027 | 36,271 | 415,775 | 30,191 |
| *Minority ethnic* |  |  |  |  |
| Without atopic eczema | 243,645 | 9,724 | 274,028 | 7,852 |
| With atopic eczema | 62,445 | 3,424 | 66,276 | 2,582 |

**Limitations**

One problem that arose when we tried to estimate the hazard ratio for the association between atopic eczema and depression in individuals from minority ethnic groups using imputed data was that omitted variables varied across imputations. In one imputation, no variables were omitted, yet in another, one or more variables were omitted. It is possible that this occurred because in some of the variable groups included in the imputation, no individuals were observed, therefore corresponding coefficients could not be estimated. Therefore, the multiple imputation of missing ethnicity data was too uncertain to carry out the Cox regression to estimate the association between atopic eczema and depression in adults from minority ethnic groups.

**Appendix S6 – Secondary analyses**

In secondary analyses, we described the proportions of total follow-up adults with atopic eczema in white and minority ethnic groups spent at each level of atopic eczema severity during follow-up and investigated whether associations between atopic eczema severity and depression or anxiety differed between individuals of white and minority ethnic groups.

**Atopic eczema severity definition**

In analyses examining atopic eczema severity, we classified individuals with atopic eczema as having mild, moderate, or severe disease using a previously developed definition.^52^ We considered individuals to have mild disease by default. We classified individuals as having moderate atopic eczema from the first of: (1) a second potent topical corticosteroid prescription within one year; or (2) a first prescription for a topical calcineurin inhibitor. We classified adults as having severe atopic eczema from the first of: (1) use of phototherapy or systemic treatment for atopic eczema (excluding systemic glucocorticoids, as they may have been prescribed for coexisting asthma); or (2) referral to a dermatologist. We updated severity over time, and once an individual was defined as having severe atopic eczema, they remained in this category for the rest of follow-up and could not be categorised as having milder disease.

**Statistical analysis**

We redefined atopic eczema exposure using atopic eczema severity. We described the proportions of total follow-up adults with atopic eczema in white and minority ethnic groups spent at each level of atopic eczema severity during follow-up. Using the same methods as the main analyses, we constructed stratified Cox regression models implicitly adjusted for matching variables (age, sex, general practice) and then sequentially adjusted for potential confounders (deprivation and calendar period) and potential mediators (comorbidity burden, comorbid asthma, harmful alcohol use, smoking status, body mass index, sleep problems and high-dose glucocorticoid use).

**References**

1. Eichenfield LF, Tom WL, Chamlin SL, et al. Guidelines of care for the management of atopic dermatitis: Part 1: Diagnosis and Assessment of Atopic Dermatitis. *J Am Acad Dermatol*. 2014;70(2):338. doi:10.1016/J.JAAD.2013.10.010

2. Kapur S, Watson W, Carr S. Atopic dermatitis. *Allergy Asthma Clin Immunol*. 2018;14(Suppl 2):52. doi:10.1186/S13223-018-0281-6

3. Chan LN, Magyari A, Ye M, et al. The epidemiology of atopic dermatitis in older adults: A population-based study in the United Kingdom. *PLoS One*. 2021;16(10). doi:10.1371/JOURNAL.PONE.0258219

4. de Lusignan S, Alexander H, Broderick C, et al. The epidemiology of eczema in children and adults in England: A population‐based study using primary care data. *Clin Exp Allergy*. 2021;51(3):471. doi:10.1111/CEA.13784

5. World Health Organisation. *Depression and Other Common Mental Disorders Global Health Estimates*. https://apps.who.int/iris/bitstream/handle/10665/254610/WHO-MSD-MER-2017.2-eng.pdf. Accessed May 18, 2022.

6. Steel Z, Marnane C, Iranpour C, et al. The global prevalence of common mental disorders: a systematic review and meta-analysis 1980–2013. *Int J Epidemiol*. 2014;43(2):476-493. doi:10.1093/IJE/DYU038

7. Rait G, Walters K, Griffin M, Buszewicz M, Petersen I, Nazareth I. Recent trends in the incidence of recorded depression in primary care. *Br J Psychiatry*. 2009;195(6):520-524. doi:10.1192/BJP.BP.108.058636

8. Ascott A, Mansfield KE, Schonmann Y, et al. Atopic eczema and obesity: a population-based study. *Br J Dermatol*. 2021;184(5):871-879. doi:10.1111/BJD.19597

9. Zhao G, Ford ES, Dhingra S, Li C, Strine TW, Mokdad AH. Depression and anxiety among US adults: associations with body mass index. *Int J Obes 2009 332*. 2009;33(2):257-266. doi:10.1038/ijo.2008.268

10. Luppino FS, De Wit LM, Bouvy PF, et al. Overweight, Obesity, and Depression: A Systematic Review and Meta-analysis of Longitudinal Studies. *Arch Gen Psychiatry*. 2010;67(3):220-229. doi:10.1001/ARCHGENPSYCHIATRY.2010.2

11. Tyrrell J, Mulugeta A, Wood AR, et al. Using genetics to understand the causal influence of higher BMI on depression. *Int J Epidemiol*. 2019;48(3):834-848. doi:10.1093/IJE/DYY223

12. Kendrick T, Stuart B, Newell C, Geraghty AWA, Moore M. Changes in rates of recorded depression in English primary care 2003-2013: Time trend analyses of effects of the economic recession, and the GP contract quality outcomes framework (QOF). *J Affect Disord*. 2015;180:68-78. doi:10.1016/j.jad.2015.03.040

13. Silverberg JI, Gelfand JM, Margolis DJ, et al. Association of atopic dermatitis with allergic, autoimmune, and cardiovascular comorbidities in US adults. *Ann Allergy Asthma Immunol*. 2018;121(5):604-612.e3. doi:10.1016/J.ANAI.2018.07.042

14. Bousquet J, Anto JM, Wickman M, et al. Are allergic multimorbidities and IgE polysensitization associated with the persistence or re-occurrence of foetal type 2 signalling? The MeDALL hypothesis. *Allergy*. 2015;70(9):1062-1078. doi:10.1111/ALL.12637

15. Aguilar D, Pinart M, Koppelman GH, et al. Computational analysis of multimorbidity between asthma, eczema and rhinitis. *PLoS One*. 2017;12(6). doi:10.1371/JOURNAL.PONE.0179125

16. Rutter CE, Silverwood RJ, Asher MI, et al. Comparison of individual-level and population-level risk factors for rhinoconjunctivitis, asthma, and eczema in the International Study of Asthma and Allergies in Childhood (ISAAC) Phase Three. *World Allergy Organ J*. 2020;13(6). doi:10.1016/J.WAOJOU.2020.100123

17. Di Marco F, Santus P, Centanni S. Anxiety and depression in asthma. *Curr Opin Pulm Med*. 2011;17(1):39-44. doi:10.1097/MCP.0B013E328341005F

18. Kwa MC, Silverberg JI. Association Between Inflammatory Skin Disease and Cardiovascular and Cerebrovascular Co-Morbidities in US Adults: Analysis of Nationwide Inpatient Sample Data. *Am J Clin Dermatol*. 2017;18(6):813-823. doi:10.1007/S40257-017-0293-X

19. Silverberg JI. Association between adult atopic dermatitis, cardiovascular disease, and increased heart attacks in three population-based studies. *Allergy*. 2015;70(10):1300-1308. doi:10.1111/ALL.12685

20. Ascott A, Mulick A, Yu AM, et al. Atopic eczema and major cardiovascular outcomes: A systematic review and meta-analysis of population-based studies. *J Allergy Clin Immunol*. 2019;143(5):1821-1829. doi:10.1016/J.JACI.2018.11.030

21. Silverberg JI, Greenland P. Eczema and cardiovascular risk factors in 2 US adult population studies. *J Allergy Clin Immunol*. 2015;135(3):721-728.e6. doi:10.1016/J.JACI.2014.11.023

22. Langan SM, Irvine AD, Weidinger S. Atopic dermatitis. *Lancet*. 2020;396(10247):345-360. doi:10.1016/S0140-6736(20)31286-1

23. Silverberg JI. Comorbidities and the impact of atopic dermatitis. *Ann Allergy, Asthma Immunol*. 2019;123(2):144-151. doi:10.1016/J.ANAI.2019.04.020

24. Lowe KE, Mansfield KE, Delmestri A, et al. Atopic eczema and fracture risk in adults: A population-based cohort study. *J Allergy Clin Immunol*. 2020;145(2):563-571.e8. doi:10.1016/J.JACI.2019.09.015

25. World Health Organization Regional Office for Europe. *Addressing Comorbidity between Mental Disorders and Major Noncommunicable Diseases*.; 2017. http://www.euro.who.int/pubrequest. Accessed May 19, 2022.

26. Chung J, Simpson EL. The socioeconomics of atopic dermatitis. *Ann Allergy, Asthma Immunol*. 2019;122:360-366. doi:10.1016/j.anai.2018.12.017

27. Edwards J, Goldie I, Elliott I, et al. *Fundamental Facts About Mental Health*.; 2016. https://www.mentalhealth.org.uk/sites/default/files/fundamental-facts-about-mental-health-2016.pdf. Accessed May 18, 2022.

28. Wamboldt MZ, Hewitt JK, Schmitz S, et al. Familial Association Between Allergic Disorders and Depression in Adult Finnish Twins. *J Med Genet (Neuro-psychiatr Genet*. 2000;96:146-153. doi:10.1002/(SICI)1096-8628(20000403)96:2

29. Al-Jefri K, Newbury-Birch D, Muirhead CR, et al. High prevalence of alcohol use disorders in patients with inflammatory skin diseases. *Br J Dermatol*. 2017;177(3):837-844. doi:10.1111/BJD.15497

30. Kathryn Mchugh R, Weiss RD. Alcohol Use Disorder and Depressive Disorders. *Alcohol Res*. 2019;40(1):e1-e8. doi:10.35946/ARCR.V40.1.01

31. Smith JP, Randall CL. Anxiety and Alcohol Use Disorders: Comorbidity and Treatment Considerations. *Alcohol Res*. 2012;34(4):414. /pmc/articles/PMC3860396/. Accessed May 19, 2022.

32. Thomsen SF. Atopic Dermatitis: Natural History, Diagnosis, and Treatment. *ISRN Allergy*. 2014;2014:1-7. doi:10.1155/2014/354250

33. Brown ES, Chandler PA. Mood and Cognitive Changes During Systemic Corticosteroid Therapy. *Prim Care Companion J Clin Psychiatry*. 2001;3(1):17. doi:10.4088/PCC.V03N0104

34. Amarbayasgalan T, Takahashi H, Dekio I, Morita E. Interleukin-8 content in the stratum corneum as an indicator of the severity of inflammation in the lesions of atopic dermatitis. *Int Arch Allergy Immunol*. 2013;160(1):63-74. doi:10.1159/000339666

35. Felger JC. Imaging the Role of Inflammation in Mood and Anxiety-related Disorders. *Curr Neuropharmacol*. 2018;16(5):533. doi:10.2174/1570159X15666171123201142

36. Simpson EL, Bieber T, Guttman-Yassky E, et al. Two Phase 3 Trials of Dupilumab versus Placebo in Atopic Dermatitis. *N Engl J Med*. 2016;375(24):2335-2348. doi:10.1056/nejmoa1610020

37. Silverberg JI. Atopic Dermatitis in Adults. doi:10.1016/j.mcna.2019.08.009

38. Johansson M, Jansson-Fröjmark M, Norell-Clarke A, Linton SJ. Changes in insomnia as a risk factor for the incidence and persistence of anxiety and depression: a longitudinal community study. *Sleep Sci Pract 2021 51*. 2021;5(1):1-9. doi:10.1186/S41606-020-00053-Z

39. Fluharty M, Taylor AE, Grabski M, Munafò MR. The Association of Cigarette Smoking With Depression and Anxiety: A Systematic Review. *Nicotine Tob Res*. 2017;19(1):3-13. doi:10.1093/NTR/NTW140

40. Yang YW, Chen YH, Huang YH. Cigarette smoking may modify the risk of depression in eczema among adults: A preliminary study using NHANES 2005-2006. *J Eur Acad Dermatology Venereol*. 2011;25(9):1048-1053. doi:10.1111/j.1468-3083.2010.03918.x

41. Roosta N, Black DS, Peng D, Riley LW. Skin disease and stigma in emerging adulthood: Impact on healthy development. *J Cutan Med Surg*. 2010;14(6):285-290. doi:10.2310/7750.2010.09053

42. Wittkowski A, Richards HL, Griffiths CEM, Main CJ. The impact of psychological and clinical factors on quality of life in individuals with atopic dermatitis. *J Psychosom Res*. 2004;57(2):195-200. doi:10.1016/S0022-3999(03)00572-5

43. Davies-Kershaw H, Petersen I, Nazareth I, Stevenson F. Factors influencing recording of drug misuse in primary care: a qualitative study of GPs in England. *Br J Gen Pract*. 2018;68(669):e234. doi:10.3399/BJGP18X695309

44. Pilz AC, Durner V, Schielein MC, et al. Addictions in patients with atopic dermatitis: a cross-sectional pilot study in Germany. *J Eur Acad Dermatology Venereol*. 2022;36(1):84-90. doi:10.1111/JDV.17708

45. Degenhardt L, Hall W, Lynskey M. Exploring the association between cannabis use and depression. *Addiction*. 2003;98(11):1493-1504. doi:10.1046/J.1360-0443.2003.00437.X

46. Davies HR, Nazareth I, Petersen I. Trends of People Using Drugs and Opioid Substitute Treatment Recorded in England and Wales General Practice (1994-2012). *PLoS One*. 2015;10(4). doi:10.1371/JOURNAL.PONE.0122626

47. Abuabara K, Magyari AM, Hoffstad O, et al. Development and Validation of an Algorithm to Accurately Identify Atopic Eczema Patients in Primary Care Electronic Health Records from the UK. *J Invest Dermatol*. 2017;137(8):1655-1662. doi:10.1016/J.JID.2017.03.029

48. Mathur R, Bhaskaran K, Chaturvedi N, et al. Completeness and usability of ethnicity data in UK-based primary care and hospital databases. *J Public Health (Bangkok)*. 2014;36(4):684-692. doi:10.1093/PUBMED/FDT116

49. Charlson M, Szatrowski TP, Peterson J, Gold J. Validation of a combined comorbidity index. *J Clin Epidemiol*. 1994;47(11):1245-1251. doi:10.1016/0895-4356(94)90129-5

50. Austin SR, Wong YN, Uzzo RG, Beck JR, Egleston BL. Why Summary Comorbidity Measures Such As the Charlson Comorbidity Index and Elixhauser Score Work. *Med Care*. 2015;53(9):e65-e72. doi:10.1097/MLR.0B013E318297429C

51. Charlson ME, Pompei P, Ales KL, MacKenzie CR. A new method of classifying prognostic comorbidity in longitudinal studies: development and validation. *J Chronic Dis*. 1987;40(5):373-383. doi:10.1016/0021-9681(87)90171-8

52. Mansfield KE, Schmidt SAJ, Darvalics B, et al. Association between Atopic Eczema and Cancer in England and Denmark. *JAMA Dermatology*. 2020;156(10):1086-1097. doi:10.1001/jamadermatol.2020.1948

Table S1: Description of sensitivity analyses, and HR (95% CI) of sensitivity analyses in depression cohort

| **Description** | **Justification** | **White ethnic group** | | | | **Minority ethnic group** | | | |
| --- | --- | --- | --- | --- | --- | --- | --- | --- | --- |
|  |  | **Number of individuals** | **PYAR** | **Events** | **HR (95% CI)** | **Number of individuals** | **PYAR** | **Events** | **HR (95% CI)** |
| **Main analysis ^a^** | | **181,173** | **724,343** | **17,246** | **1.15 (1.12, 1.17)** | **31,327** | **109,442** | **1,919** | **1.33 (1.22, 1.45)** |
| Repeating the main analysis using alternative code lists to identify depression outcome (including broader codes and symptom codes) | To explore the sensitivity of the results due to the definitions of the depression outcome | 180,499 | 721,162 | 17,282 | 1.14 (1.12, 1.17) | 31,263 | 109,158 | 1,942 | 1.35 (1.23, 1.47) |
| Restricting cohort entry to individuals with at least one consultation with their GP in the year before cohort entry. | To exclude individuals who are practice non-attenders. There may be differential recording of exposure, covariates and outcomes among practice attenders and non-attenders. For example, practice non-attenders may be more likely to have missing smoking or BMI data. | 123,501 | 477,160 | 12,192 | 1.08 (1.06, 1.11) | 22,453 | 77,253 | 1,431 | 1.22 (1.10, 1.36) |
| Repeating the main analysis after removing censoring at the time of an alternative diagnoses that may also represent the outcome of interest (i.e., severe mental illness). | To avoid potentially informative censoring of outcomes by severe mental illness. | 181,173 | 724,343 | 17,246 | 1.15 (1.12, 1.17) | 31,327 | 109,442 | 1,919 | 1.33 (1.22, 1.45) |
| Repeating the main analysis using a redefined cohort of adults entering from 1 April 2006 that are eligible for linkage with HES and have complete ethnicity data. | To explore the sensitivity of our results to the definition of our study population and examine whether the study population of the main analysis is susceptible to selection bias. The main study population included only individuals with complete ethnicity data who are likely to be different from those with missing ethnicity data, which may introduce selection bias. Previous work has shown that combining CPRD and HES increases the completeness of ethnicity data. | 97,565 | 371,235 | 8,850 | 1.13 (1.10, 1.16) | 20,821 | 71,221 | 1,271 | 1.33 (1.20, 1.47) |
| Repeating the main analysis using a redefined cohort of adults entering from 1 April 2006. Missing ethnicity data was imputed using multiple imputation. ^b^ | To explore the sensitivity of our results to the definition of our study population and examine whether the study population of the main analysis is susceptible to selection bias. | n/a | n/a | n/a | 1.20 (1.19,1.22) | n/a | n/a | n/a | n/a |
| **Main analysis further adjusted for potential mediators ^c^** | | **145,302** | **604,767** | **14,436** | **1.05 (1.03, 1.08)** | **25,478** | **93,033** | **1,665** | **1.14 (1.02, 1.26)** |
| Repeating the main analysis using less strict definitions for sleep problems (main analysis code list includes Zolpidem and Zopiclone which are only prescribed for sleep problems, sensitivity analysis code list expanded to include prescriptions for benzodiazepines, melatonin, and other drugs). | To explore whether including broader drugs that are prescribed for conditions other than sleep disturbances further mediates the association between atopic eczema and severe mental illness. | 145,302 | 604,767 | 14,436 | 1.04 (1.02, 1.07) | 25,478 | 93,033 | 1,665 | 1.12 (1.01, 1.25) |

Abbreviations: CI – Confidence Interval; HR – Hazard Ratio; PYAR – Person years at risk

^a^ Adjusted for calendar time and quintiles of Carstairs deprivation index (using 2011 census data)

^b^ Effect estimate from cohort of 427,366 people with atopic eczema matched to 1,699,374 without. 1,099,210 individuals in the cohort had complete ethnicity data (939,135 from the white ethnic group, 160,075 from the minority ethnic group) and 990,980 had missing ethnicity data which was imputed. During follow-up, there were 101,538 depression events. The number of observations among the subpopulations of people from white and minority ethnic groups varied across imputations, therefore numbers of individuals, PYARs and numbers of events could not be obtained. Producing an effect estimate for the minority ethnic group was not feasible as omitted variables varied across imputations.

^c^ Cohorts are further adjusted for comorbidity burden (using the Charlson comorbidity index), comorbid asthma, sleep problems, smoking status, high dose glucocorticoid use, harmful alcohol use and body mass index

Table S2: Description of sensitivity analyses, and HR (95% CI) of sensitivity analyses in anxiety cohort

| **Description** | **Justification** | **White ethnic group** | | | | **Minority ethnic group** | | | |
| --- | --- | --- | --- | --- | --- | --- | --- | --- | --- |
|  |  | **Number of individuals** | **PYAR** | **Events** | **HR (95% CI)** | **Number of individuals** | **PYAR** | **Events** | **HR (95% CI)** |
| **Main analysis ^a^** |  | **205,779** | **836,483** | **15,080** | **1.17 (1.14,1.19)** | **33,995** | **120,072** | **1,501** | **1.41 (1.28,1.55)** |
| Repeating the main analysis using alternative code lists to identify depression and anxiety outcomes (including broader codes and symptom codes) | To explore the sensitivity of the results due to the definitions of the depression and anxiety outcomes | 201,012 | 814,678 | 15,883 | 1.17 (1.15,1.20) | 33,266 | 117,406 | 1,735 | 1.41 (1.29, 1.54) |
| Restricting cohort entry to individuals with at least one consultation with their GP in the year before cohort entry. | To exclude individuals who are practice non-attenders. There may be differential recording of exposure, covariates and outcomes among practice attenders and non-attenders. For example, practice non-attenders may be more likely to have missing smoking or BMI data. | 141,768 | 556,742 | 10,809 | 1.11 (1.08, 1.14) | 24,529 | 85,617 | 1,085 | 1.31 (1.16, 1.48) |
| Repeating the main analysis after removing censoring at the time of an alternative diagnoses that may also represent the outcome of interest (i.e., severe mental illness). | To avoid potentially informative censoring of outcomes by severe mental illness. | 205,779 | 836,483 | 15,080 | 1.17 (1.14, 1.19) | 33,995 | 120,702 | 1,501 | 1.41 (1.28, 1.55) |
| Repeating the main analysis using a redefined cohort of adults entering from 1 April 2006 that are eligible for linkage with HES and have complete ethnicity data. | To explore the sensitivity of our results to the definition of our study population and examine whether the study population of the main analysis is susceptible to selection bias. The main study population included only individuals with complete ethnicity data who are likely to be different from those with missing ethnicity data, which may introduce selection bias. Previous work has shown that combining CPRD and HES increases the completeness of ethnicity data. | 111,237 | 429,021 | 7,645 | 1.17 (1.13, 1.20) | 22,780 | 79,030 | 1,037 | 1.42 (1.27, 1.59) |
| Repeating the main analysis using a redefined cohort of adults entering from 1 April 2006. Missing ethnicity data was imputed using multiple imputation. ^b^ | To explore the sensitivity of our results to the definition of our study population and examine whether the study population of the main analysis is susceptible to selection bias. | n/a | n/a | n/a | 1.22 (1.20, 1.23) | n/a | n/a | n/a | 1.38 (1.25, 1.52) |
| **Main analysis further adjusted for mediators ^d^** | | **168,344** | **710,076** | **12,849** | **1.07 (1.04, 1.09)** | **27,963** | **103,522** | **1,322** | **1.22 (1.09, 1.37)** |
| Repeating the main analysis using less strict definitions for sleep problems (main analysis code list includes Zolpidem and Zopiclone which are only prescribed for sleep problems, sensitivity analysis code list expanded to include prescriptions for benzodiazepines, melatonin, and other drugs). | To explore whether including broader drugs that are prescribed for conditions other than sleep disturbances further mediates the association between atopic eczema and severe mental illness. | 168,344 | 710,076 | 12,849 | 1.03 (1.01, 1.06) | 27,963 | 103,522 | 1,322 | 1.15 (1.03, 1.30) |

Abbreviations: CI – Confidence Interval; HR – Hazard Ratio; PYAR – Person years at risk

^a^ Adjusted for calendar time and quintiles of Carstairs deprivation index (using 2011 census data)

^b^ Effect estimates from cohort of 471,860 people with atopic eczema matched to 1,991,567 without. 1,292,737 individuals in the cohort had complete ethnicity data (1,112,495 from the white ethnic group, 180,242 from the minority ethnic group) and 1,125,489 had missing ethnicity data which was imputed. During follow-up, there were 82,367 anxiety events. The number of observations among the subpopulations of people from white and minority ethnic groups varied across imputations, therefore numbers of individuals, PYARs and numbers of events could not be obtained.

^c^ Cohorts are further adjusted for comorbidity burden (using the Charlson comorbidity index), comorbid asthma, sleep problems, smoking status, high dose glucocorticoid use, harmful alcohol use and body mass index

Table S3: Characteristics of main analysis (confounder-adjusted) cohort, HES-enriched sensitivity cohort, and multiple imputation of missing ethnicity cohort used to investigate associations between atopic eczema and depression in white and minority ethnic groups

|  | **Main analysis cohort** | | **HES-enriched cohort** | | **Multiple imputation of missing ethnicity cohort** | |
| --- | --- | --- | --- | --- | --- | --- |
|  | **With atopic eczema** | **Without atopic eczema** | **With atopic eczema** | **Without atopic eczema** | **With atopic eczema** | **Without atopic eczema** |
|  | n=212,500 | n=639,470 | n=118,386 | n=339,243 | n=427,366 | n=1,699,374 |
| **Follow-up ^a^** |  |  |  |  |  |  |
| Total person-years | 833,785 | 2,350,951 | 442,456 | 1,206,507 | 1,757,814 | 6,724,293 |
| Median (IQR) duration of follow-up (years) | 3.2 (1.3-5.9) | 2.8 (1.2-5.5) | 3.0 (1.3-5.6) | 2.8 (1.2-5.3) | 3.3 (1.3-6.3) | 3.1 (1.2-6.1) |
| **Sex** |  |  |  |  |  |  |
| Female (%) | 117,663 (55.4%) | 350,103 (54.7%) | 66,540 (56.2%) | 191,075 (56.3%) | 226,360 (53.0%) | 850,026 (50.0%) |
| **Age (years) ^b^** |  |  |  |  |  |  |
| 18-29 | 82,012 (38.6%) | 264,086 (41.3%) | 43,693 (36.9%) | 136,920 (40.4%) | 179,947 (42.1%) | 769,430 (45.3%) |
| 30-39 | 36,122 (17.0%) | 112,161 (17.5%) | 19,612 (16.6%) | 55,633 (16.4%) | 59,297 (13.9%) | 239,124 (14.1%) |
| 40-49 | 25,234 (11.9%) | 69,899 (10.9%) | 14,157 (12.0%) | 35,749 (10.5%) | 47,022 (11.0%) | 176,046 (10.4%) |
| 50-59 | 21,397 (10.1%) | 58,833 (9.2%) | 12,189 (10.3%) | 31,767 (9.4%) | 42,192 (9.9%) | 156,222 (9.2%) |
| 60-69 | 21,837 (10.3%) | 62,606 (9.8%) | 13,077 (11.0%) | 36,359 (10.7%) | 43,054 (10.1%) | 162,543 (9.6%) |
| 70+ | 25,898 (12.2%) | 71,885 (11.2%) | 15,658 (13.2%) | 42,815 (12.6%) | 55,854 (13.1%) | 196,009 (11.5%) |
| **Ethnicity** |  |  |  |  |  |  |
| White | 181,173 (85.3%) | 541,478 (84.7%) | 97,565 (82.4%) | 279,304 (82.3%) | 196,927 (46.1%) | 758,847 (44.7%) |
| Minority ethnic | 31,327 (14.7%) | 97,992 (15.3%) | 20,821 (17.6%) | 59,939 (17.7%) | 32,872 (7.7%) | 130,373 (7.7%) |
| Missing | n/a | n/a | n/a | n/a | 197,567 (46.2%) | 810,154 (47.7%) |
| **Quintiles of Carstairs deprivation index ^c^** |  |  |  |  |  |  |
| 1 - Least deprived | 40,005 (18.8%) | 112,671 (17.6%) | 28,286 (23.9%) | 76,485 (22.5%) | 84,902 (19.9%) | 327,853 (19.3%) |
| 2 | 42,331 (19.9%) | 127,095 (19.9%) | 25,821 (21.8%) | 72,202 (21.3%) | 84,163 (19.7%) | 330,950 (19.5%) |
| 3 | 44,016 (20.7%) | 129,585 (20.3%) | 23,394 (19.8%) | 65,002 (19.2%) | 92,188 (21.6%) | 364,776 (21.5%) |
| 4 | 45,293 (21.3%) | 138,247 (21.6%) | 21,710 (18.3%) | 64,214 (18.9%) | 94,856 (22.2%) | 380,465 (22.4%) |
| 5 - Most deprived | 40,855 (19.2%) | 131,872 (20.6%) | 19,175 (16.2%) | 61,340 (18.1%) | 71,257 (16.7%) | 295,330 (17.4%) |
| Missing | n/a | n/a | n/a | n/a | n/a | n/a |
| **Body mass index (kg/m^2^) ^d^** |  |  |  |  |  |  |
| Underweight (<18.5) | 5,525 (2.6%) | 18,766 (2.9%) | 3,187 (2.7%) | 10,223 (3.0%) | 10,901 (2.6%) | 44,914 (2.6%) |
| Normal (18.5-24.9) | 77,544 (36.5%) | 230,252 (36.0%) | 45,059 (38.1%) | 125,858 (37.1%) | 144,072 (33.7%) | 530,546 (31.2%) |
| Overweight (25-29.9) | 57,035 (26.8%) | 161,216 (25.2%) | 32,544 (27.5%) | 86,966 (25.6%) | 106,064 (24.8%) | 376,390 (22.1%) |
| Obese (30+) | 38,378 (18.1%) | 103,913 (16.2%) | 20,715 (17.5%) | 55,011 (16.2%) | 71,336 (16.7%) | 242,370 (14.3%) |
| Missing | 34,018 (16.0%) | 125,323 (19.6%) | 16,881 (14.3%) | 61,185 (18.0%) | 94,993 (22.2%) | 505,154 (29.7%) |
| **Smoking status ^d^** |  |  |  |  |  |  |
| Non-smoker | 114,066 (53.7%) | 342,819 (53.6%) | 63,445 (53.6%) | 181,253 (53.4%) | 232,869 (54.5%) | 876,966 (51.6%) |
| Current or ex-smoker | 94,525 (44.5%) | 275,352 (43.1%) | 53,184 (44.9%) | 148,145 (43.7%) | 178,971 (41.9%) | 666,808 (39.2%) |
| Missing | 3,909 (1.8%) | 21,299 (3.3%) | 1,757 (1.5%) | 9,845 (2.9%) | 15,526 (3.6%) | 155,600 (9.2%) |
| **Charlson Comorbidity Index ^d^** |  |  |  |  |  |  |
| Low (0) | 132,686 (62.4%) | 474,241 (74.2%) | 74,310 (62.8%) | 248,889 (73.4%) | 269,200 (63.0%) | 1,270,184 (74.7%) |
| Moderate (1-2) | 70,731 (33.3%) | 137,779 (21.5%) | 38,977 (32.9%) | 74,532 (22.0%) | 140,414 (32.9%) | 360,088 (21.2%) |
| Severe (3 or more) | 9,083 (4.3%) | 27,450 (4.3%) | 5,099 (4.3%) | 15,822 (4.7%) | 17,752 (4.2%) | 69,102 (4.1%) |
| **Asthma (%) ^d^** | 54,194 (25.5%) | 81,832 (12.8%) | 29,654 (25.0%) | 43,618 (12.9%) | 108,226 (25.3%) | 221,362 (13.0%) |
| **Harmful alcohol use (%) ^d^** | 15,733 (7.4%) | 39,803 (6.2%) | 8,088 (6.8%) | 19,180 (5.7%) | 27,200 (6.4%) | 88,970 (5.2%) |
| **Problems with sleep (%) ^d^** | 36,757 (17.3%) | 65,742 (10.3%) | 20,381 (17.2%) | 35,326 (10.4%) | 73,874 (17.3%) | 169,342 (10.0%) |

Abbreviations: IQR: Interquartile range

Individuals can contribute data as both atopic eczema exposed and unexposed. Therefore, numbers of exposed/unexposed do not total the whole cohort, as individuals may be included in more than one column.

^a^ Follow-up based on censoring at the earliest of: death, no longer registered with practice, practice no longer contributing to CPRD, or depression or anxiety diagnosis, diagnosis that suggests an alternative cause of the depression or anxiety outcome (severe mental illness)

^b^ Age at index date

^c^ Carstairs deprivation index based on practice-level data (from 2011).

^d^ Based on records closest to index date.

|  | | |  | |  |  |  |  |  |  |
| --- | --- | --- | --- | --- | --- | --- | --- | --- | --- | --- |
|  |  |  | |  |  |  |  |  |  |  |
|  | | |  | |  |  |  |  |  |  |
|  | | |  | |  |  |  |  |  |  |
|  | | |  | |  |  |  |  |  |  |

Table S4: Characteristics of main analysis (confounder-adjusted) cohort, HES-enriched sensitivity cohort, and multiple imputation of missing ethnicity cohort used to investigate associations between atopic eczema and anxiety in white and minority ethnic groups

|  | **Main analysis cohort** | | **HES-enriched cohort** | | **Multiple imputation of missing ethnicity cohort** | |
| --- | --- | --- | --- | --- | --- | --- |
|  | **With atopic eczema** | **Without atopic eczema** | **With atopic eczema** | **Without atopic eczema** | **With atopic eczema** | **Without atopic eczema** |
|  | n=239,774 | n=765,991 | n=134,017 | n=407,680 | n=471,860 | n=1,991,567 |
| **Follow-up ^a^** |  |  |  |  |  |  |
| Total person-years | 957,185 | 2,861,456 | 508,051 | 1,471,796 | 1,977,734 | 8,013,905 |
| Median (IQR) duration of follow-up (years) | 3.2 (1.4-6.0) | 2.9 (1.2-5.6) | 3.1 (1.3-5.7) | 2.9 (1.2-5.4) | 3.4 (1.4-6.4) | 3.2 (1.3-6.2) |
| **Sex** |  |  |  |  |  |  |
| Female (%) | 137,304 (57.3%) | 441,348 (57.6%) | 78,014 (58.2%) | 242,305 (59.4%) | 258,395 (54.8%) | 1,052,359 (52.8%) |
| **Age (years) ^b^** |  |  |  |  |  |  |
| 18-29 | 87,674 (36.6%) | 293,892 (38.4%) | 46,913 (35.0%) | 152,832 (37.5%) | 188,677 (40.0%) | 833,089 (41.8%) |
| 30-39 | 41,633 (17.4%) | 138,644 (18.1%) | 22,673 (16.9%) | 69,111 (17.0%) | 67,674 (14.3%) | 295,620 (14.8%) |
| 40-49 | 30,814 (12.9%) | 93,953 (12.3%) | 17,254 (12.9%) | 48,076 (11.8%) | 55,897 (11.8%) | 232,047 (11.7%) |
| 50-59 | 25,845 (10.8%) | 77,856 (10.2%) | 14,692 (11.0%) | 41,871 (10.3%) | 49,745 (10.5%) | 203,296 (10.2%) |
| 60-69 | 25,056 (10.4%) | 76,975 (10.0%) | 15,017 (11.2%) | 44,918 (11.0%) | 48,490 (10.3%) | 198,155 (9.9%) |
| 70+ | 28,752 (12.0%) | 84,671 (11.1%) | 17,468 (13.0%) | 50,872 (12.5%) | 61,377 (13.0%) | 229,360 (11.5%) |
| **Ethnicity** |  |  |  |  |  |  |
| White | 205,779 (85.8%) | 653,373 (85.3%) | 111,237 (83.0%) | 338,617 (83.1%) | 221,343 (46.9%) | 912,119 (45.8%) |
| Minority ethnic | 33,995 (14.2%) | 112,618 (14.7%) | 22,780 (17.0%) | 69,063 (16.9%) | 35,391 (7.5%) | 148,682 (7.5%) |
| Missing | n/a | n/a | n/a | n/a | 215,126 (45.6%) | 930,766 (46.7%) |
| **Quintiles of Carstairs deprivation index ^c^** |  |  |  |  |  |  |
| 1 - Least deprived | 44,123 (18.4%) | 132,325 (17.3%) | 31,175 (23.3%) | 90,160 (22.1%) | 91,748 (19.4%) | 376,606 (18.9%) |
| 2 | 46,993 (19.6%) | 149,716 (19.5%) | 28,860 (21.5%) | 85,926 (21.1%) | 91,895 (19.5%) | 384,119 (19.3%) |
| 3 | 49,443 (20.6%) | 154,764 (20.2%) | 26,606 (19.9%) | 79,015 (19.4%) | 101,479 (21.5%) | 425,502 (21.4%) |
| 4 | 52,153 (21.8%) | 168,516 (22.0%) | 25,108 (18.7%) | 78,421 (19.2%) | 106,617 (22.6%) | 455,574 (22.9%) |
| 5 - Most deprived | 47,062 (19.6%) | 160,670 (21.0%) | 22,268 (16.6%) | 74,158 (18.2%) | 80,121 (17.0%) | 349,766 (17.6%) |
| Missing | n/a | n/a | n/a | n/a | n/a | n/a |
| **Body mass index (kg/m^2^) ^d^** |  |  |  |  |  |  |
| Underweight (<18.5) | 6,093 (2.5%) | 21,951 (2.9%) | 3,513 (2.6%) | 11,991 (2.9%) | 11,874 (2.5%) | 52,060 (2.6%) |
| Normal (18.5-24.9) | 85,862 (35.8%) | 273,573 (35.7%) | 50,059 (37.4%) | 150,246 (36.9%) | 157,296 (33.3%) | 625,197 (31.4%) |
| Overweight (25-29.9) | 64,917 (27.1%) | 196,450 (25.6%) | 37,138 (27.7%) | 106,273 (26.1%) | 118,764 (25.2%) | 454,555 (22.8%) |
| Obese (30+) | 47,000 (19.6%) | 135,493 (17.7%) | 25,463 (19.0%) | 71,842 (17.6%) | 85,491 (18.1%) | 312,146 (15.7%) |
| Missing | 35,902 (15.0%) | 138,524 (18.1%) | 17,844 (13.3%) | 67,328 (16.5%) | 98,435 (20.9%) | 547,609 (27.5%) |
| **Smoking status ^d^** |  |  |  |  |  |  |
| Non-smoker | 123,333 (51.4%) | 395,175 (51.6%) | 68,824 (51.4%) | 210,325 (51.6%) | 247,432 (52.4%) | 996,610 (50.0%) |
| Current or ex-smoker | 112,504 (46.9%) | 348,409 (45.5%) | 63,430 (47.3%) | 186,955 (45.9%) | 208,804 (44.3%) | 830,528 (41.7%) |
| Missing | 3,937 (1.6%) | 22,407 (2.9%) | 1,763 (1.3%) | 10,400 (2.6%) | 15,624 (3.3%) | 164,429 (8.3%) |
| **Charlson Comorbidity Index ^d^** |  |  |  |  |  |  |
| Low (0) | 147,185 (61.4%) | 559,610 (73.1%) | 82,611 (61.6%) | 294,593 (72.3%) | 292,987 (62.1%) | 1,467,740 (73.7%) |
| Moderate (1-2) | 81,550 (34.0%) | 170,920 (22.3%) | 45,130 (33.7%) | 92,684 (22.7%) | 157,614 (33.4%) | 435,448 (21.9%) |
| Severe (3 or more) | 11,039 (4.6%) | 35,461 (4.6%) | 6,276 (4.7%) | 20,403 (5.0%) | 21,259 (4.5%) | 88,379 (4.4%) |
| **Asthma (%) ^d^** | 62,501 (26.1%) | 101,698 (13.3%) | 34,389 (25.7%) | 54,533 (13.4%) | 121,442 (25.7%) | 265,742 (13.3%) |
| **Harmful alcohol use (%) ^d^** | 19,484 (8.1%) | 52,503 (6.9%) | 9,980 (7.4%) | 25,231 (6.2%) | 32,870 (7.0%) | 115,723 (5.8%) |
| **Problems with sleep (%) ^d^** | 46,876 (19.6%) | 91,862 (12.0%) | 26,193 (19.5%) | 49,507 (12.1%) | 91,154 (19.3%) | 228,679 (11.5%) |

Abbreviations: IQR: Interquartile range

Individuals can contribute data as both atopic eczema exposed and unexposed. Therefore, numbers of exposed/unexposed do not total the whole cohort, as individuals may be included in more than one column.

^a^ Follow-up based on censoring at the earliest of: death, no longer registered with practice, practice no longer contributing to CPRD, or depression or anxiety diagnosis, diagnosis that suggests an alternative cause of the depression or anxiety outcome (severe mental illness)

^b^ Age at index date

^c^ Carstairs deprivation index based on practice-level data (from 2011).

^d^ Based on records closest to index date.

|  | | |  | |  |  |  |  |  |  |
| --- | --- | --- | --- | --- | --- | --- | --- | --- | --- | --- |
|  |  |  | |  |  |  |  |  |  |  |
|  | | |  | |  |  |  |  |  |  |
|  | | |  | |  |  |  |  |  |  |
|  | | |  | |  |  |  |  |  |  |

Table S5: Comparison of baseline characteristics of individuals with recorded and without recorded ethnicity in depression and anxiety cohorts

|  | **Depression cohort** | | **Anxiety cohort** | |
| --- | --- | --- | --- | --- |
|  | **With recorded ethnicity** | **Without recorded ethnicity** | **With recorded ethnicity** | **Without recorded ethnicity** |
|  | n=1,117,355 | n=1,046,043 | n=1,313,076 | n=1,185,642 |
| **Follow-up ^a^** |  |  |  |  |
| Total person-years | 4,262,859 | 4,600,242 | 5,100,510 | 5,318,617 |
| Median (IQR) duration of follow-up (years) | 3.0 (1.2-5.8) | 3.5 (1.4-6.8) | 3.1 (1.2-5.9) | 3.6 (1.4-7.0) |
| **Sex** |  |  |  |  |
| Female (%) | 601,991 (53.9%) | 489,905 (46.8%) | 742,074 (56.5%) | 582,216 (49.1%) |
| **Age (years) ^b^** |  |  |  |  |
| 18-29 | 471,845 (42.2%) | 505,950 (48.4%) | 517,033 (39.4%) | 532,712 (44.9%) |
| 30-39 | 184,357 (16.5%) | 120,211 (11.5%) | 224,371 (17.1%) | 145,522 (12.3%) |
| 40-49 | 122,802 (11.0%) | 104,533 (10.0%) | 160,302 (12.2%) | 132,388 (11.2%) |
| 50-59 | 104,636 (9.4%) | 97,270 (9.3%) | 135,206 (10.3%) | 121,945 (10.3%) |
| 60-69 | 110,258 (9.9%) | 97,271 (9.3%) | 133,469 (10.2%) | 115,292 (9.7%) |
| 70+ | 127,737 (11.4%) | 124,285 (11.9%) | 148,280 (11.3%) | 142,295 (12.0%) |
| **Quintiles of Carstairs deprivation index ^c^** |  |  |  |  |
| 1 - Least deprived | 204,505 (18.3%) | 200,770 (19.2%) | 235,498 (17.9%) | 223,927 (18.9%) |
| 2 | 215,813 (19.3%) | 192,460 (18.4%) | 250,294 (19.1%) | 217,285 (18.3%) |
| 3 | 225,897 (20.2%) | 223,174 (21.3%) | 264,902 (20.2%) | 252,475 (21.3%) |
| 4 | 239,325 (21.4%) | 227,728 (21.8%) | 286,912 (21.9%) | 264,733 (22.3%) |
| 5 - Most deprived | 213,670 (19.1%) | 146,848 (14.0%) | 255,131 (19.4%) | 167,069 (14.1%) |
| Missing | 18,145 (1.6%) | 55,063 (5.3%) | 20,339 (1.5%) | 60,153 (5.1%) |
| **Body mass index (kg/m^2^) ^d^** |  |  |  |  |
| Underweight (<18.5) | 32,106 (2.9%) | 24,896 (2.4%) | 36,922 (2.8%) | 28,049 (2.4%) |
| Normal (18.5-24.9) | 399,001 (35.7%) | 287,409 (27.5%) | 463,892 (35.3%) | 329,822 (27.8%) |
| Overweight (25-29.9) | 281,072 (25.2%) | 207,016 (19.8%) | 335,834 (25.6%) | 242,919 (20.5%) |
| Obese (30+) | 182,403 (16.3%) | 134,097 (12.8%) | 233,195 (17.8%) | 166,751 (14.1%) |
| Missing | 222,773 (19.9%) | 392,625 (37.5%) | 243,233 (18.5%) | 418,101 (35.3%) |
| **Smoking status ^d^** |  |  |  |  |
| Non-smoker | 599,716 (53.7%) | 532,612 (50.9%) | 677,967 (51.6%) | 587,371 (49.5%) |
| Current or ex-smoker | 479,678 (42.9%) | 376,239 (36.0%) | 595,641 (45.4%) | 453,501 (38.2%) |
| Missing | 37,961 (3.4%) | 137,192 (13.1%) | 39,468 (3.0%) | 144,770 (12.2%) |
| **Charlson Comorbidity Index ^d^** |  |  |  |  |
| Low (0) | 800,837 (71.7%) | 768,267 (73.4%) | 928,109 (70.7%) | 861,811 (72.7%) |
| Moderate (1-2) | 268,616 (24.0%) | 238,568 (22.8%) | 324,002 (24.7%) | 274,957 (23.2%) |
| Severe (3 or more) | 47,902 (4.3%) | 39,208 (3.7%) | 60,965 (4.6%) | 48,874 (4.1%) |
| **Asthma (%) ^d^** | 173,385 (15.5%) | 161,288 (15.4%) | 208,214 (15.9%) | 183,239 (15.5%) |
| **Harmful alcohol use (%) ^d^** | 72,132 (6.5%) | 44,755 (4.3%) | 93,286 (7.1%) | 56,014 (4.7%) |
| **Problems with sleep (%) ^d^** | 129,887 (11.6%) | 119,421 (11.4%) | 175,080 (13.3%) | 150,706 (12.7%) |

Abbreviations: IQR: Interquartile range

Individuals can contribute data as both atopic eczema exposed and unexposed. Therefore, numbers of exposed/unexposed do not total the whole cohort, as individuals may be included in more than one column.

^a^ Follow-up based on censoring at the earliest of: death, no longer registered with practice, practice no longer contributing to CPRD, or depression or anxiety diagnosis, diagnosis that suggests an alternative cause of the depression or anxiety outcome (severe mental illness)

^b^ Age at index date

^c^ Carstairs deprivation index based on practice-level data (from 2011).

^d^ Based on records closest to index date.

Table S6: Person-time under follow-up in depression and anxiety cohorts broken down by individual-level characteristics and atopic eczema exposure status

|  | **Depression cohort** | | **Anxiety cohort** | |
| --- | --- | --- | --- | --- |
|  | **With atopic eczema** | **Without atopic eczema** | **With atopic eczema** | **Without atopic eczema** |
|  | n=215,073 | n=646,539 | n=242,598 | n=774,113 |
| **Follow-up ^a^** |  |  |  |  |
| Total person-years | 845,534 | 2,381,779 | 970,230 | 2,898,227 |
| Median (IQR) duration of follow-up (years) | 3.2 (1.3-5.9) | 2.8 (1.2-5.5) | 3.3 (1.4-6.0) | 2.9 (1.2-5.6) |
| **Sex** |  |  |  |  |
| Female (%) | 459,129 (54.3%) | 1,260,967 (52.9%) | 547,861 (56.5%) | 1,628,566 (56.2%) |
| **Age (years) ^b^** |  |  |  |  |
| 18-29 | 281,931 (33.3%) | 842,812 (35.4%) | 308,702 (31.8%) | 956,354 (33.0%) |
| 30-39 | 142,008 (16.8%) | 403,118 (16.9%) | 167,164 (17.2%) | 509,384 (17.6%) |
| 40-49 | 113,254 (13.4%) | 298,536 (12.5%) | 140,056 (14.4%) | 405,274 (14.0%) |
| 50-59 | 98,913 (11.7%) | 264,508 (11.1%) | 119,707 (12.3%) | 347,775 (12.0%) |
| 60-69 | 105,224 (12.4%) | 292,866 (12.3%) | 119,819 (12.3%) | 353,903 (12.2%) |
| 70+ | 104,204 (12.3%) | 279,939 (11.8%) | 114,783 (11.8%) | 325,536 (11.2%) |
| **Ethnicity** |  |  |  |  |
| White | 735,495 (87.0%) | 2,083,588 (87.5%) | 848,896 (87.5%) | 2,550,372 (88.0%) |
| Minority ethnic | 110,039 (13.0%) | 298,191 (12.5%) | 121,334 (12.5%) | 347,855 (12.0%) |
| **Quintiles of Carstairs deprivation index ^c^** |  |  |  |  |
| 1 - Least deprived | 156,862 (18.6%) | 420,347 (17.6%) | 174,640 (18.0%) | 498,743 (17.2%) |
| 2 | 160,296 (19.0%) | 451,953 (19.0%) | 180,086 (18.6%) | 539,198 (18.6%) |
| 3 | 174,334 (20.6%) | 473,431 (19.9%) | 198,407 (20.4%) | 573,725 (19.8%) |
| 4 | 181,471 (21.5%) | 522,120 (21.9%) | 213,193 (22.0%) | 645,682 (22.3%) |
| 5 - Most deprived | 160,821 (19.0%) | 483,100 (20.3%) | 190,859 (19.7%) | 604,108 (20.8%) |
| Missing | 11,749 (1.4%) | 30,828 (1.3%) | 13,045 (1.3%) | 36,771 (1.3%) |
| **Body mass index (kg/m^2^) ^d^** |  |  |  |  |
| Underweight (<18.5) | 19,656 (2.3%) | 60,532 (2.5%) | 22,108 (2.3%) | 72,028 (2.5%) |
| Normal (18.5-24.9) | 309,831 (36.6%) | 845,639 (35.5%) | 347,158 (35.8%) | 1,020,088 (35.2%) |
| Overweight (25-29.9) | 244,152 (28.9%) | 657,207 (27.6%) | 280,832 (28.9%) | 807,041 (27.8%) |
| Obese (30+) | 162,093 (19.2%) | 425,212 (17.9%) | 202,625 (20.9%) | 561,062 (19.4%) |
| Missing | 109,802 (13.0%) | 393,189 (16.5%) | 117,508 (12.1%) | 438,008 (15.1%) |
| **Smoking status ^d^** |  |  |  |  |
| Non-smoker | 460,175 (54.4%) | 1,287,472 (54.1%) | 504,246 (52.0%) | 1,503,145 (51.9%) |
| Current or ex-smoker | 378,148 (44.7%) | 1,046,599 (43.9%) | 458,665 (47.3%) | 1,344,154 (46.4%) |
| Missing | 7,212 (0.9%) | 47,707 (2.0%) | 7,320 (0.8%) | 50,928 (1.8%) |
| **Charlson Comorbidity Index ^d^** |  |  |  |  |
| Low (0) | 537,885 (63.6%) | 1,760,084 (73.9%) | 606,702 (62.5%) | 2,113,349 (72.9%) |
| Moderate (1-2) | 274,688 (32.5%) | 527,027 (22.1%) | 323,575 (33.4%) | 661,722 (22.8%) |
| Severe (3 or more) | 32,961 (3.9%) | 94,667 (4.0%) | 39,953 (4.1%) | 123,156 (4.2%) |
| **Calendar time** |  |  |  |  |
| 2006-2010 | 150,033 (17.7%) | 423,453 (17.8%) | 169,427 (17.5%) | 504,116 (17.4%) |
| 2011-2015 | 425,043 (50.3%) | 1,207,024 (50.7%) | 489,255 (50.4%) | 1,475,157 (50.9%) |
| 2016-2020 | 270,459 (32.0%) | 751,301 (31.5%) | 311,548 (32.1%) | 918,955 (31.7%) |
| **Asthma (%) ^d^** | 210,868 (24.9%) | 313,723 (13.2%) | 249,392 (25.7%) | 398,170 (13.7%) |
| **Harmful alcohol use (%) ^d^** | 70,167 (8.3%) | 173,203 (7.3%) | 88,514 (9.1%) | 231,232 (8.0%) |
| **Problems with sleep (%) ^d^** | 157,538 (18.6%) | 273,866 (11.5%) | 206,138 (21.2%) | 393,992 (13.6%) |
| **High-dose oral glucocorticoids (20mg+ prednisolone equivalent dose)** | 58,946 (7.0%) | 125,768 (5.3%) | 72,272 (7.4%) | 165,533 (5.7%) |

Abbreviations: IQR: Interquartile range

Individuals can contribute data as both atopic eczema exposed and unexposed. Therefore, numbers of exposed/unexposed do not total the whole cohort, as individuals may be included in more than one column.

^a^ Follow-up based on censoring at the earliest of: death, no longer registered with practice, practice no longer contributing to CPRD, or depression or anxiety diagnosis, diagnosis that suggests an alternative cause of the depression or anxiety outcome (severe mental illness)

^b^ Age at index date

^c^ Carstairs deprivation index based on practice-level data (from 2011).

^d^ Based on records closest to index date.

|  | | |  | |  |  |  |  |  |  |
| --- | --- | --- | --- | --- | --- | --- | --- | --- | --- | --- |
|  |  |  | |  |  |  |  |  |  |  |
|  | | |  | |  |  |  |  |  |  |
|  | | |  | |  |  |  |  |  |  |
|  | | |  | |  |  |  |  |  |  |

Table S7: Characteristics of the depression cohort at cohort entry, for: the overall cohort, individuals included in the model additionally adjusting for potential confounders (i.e., individuals with no missing Carstairs deprivation data), individuals with missing Carstairs data, individuals included in the model additionally adjusting for potential mediators (i.e., individuals with no missing BMI or smoking status data), and for individuals with missing BMI or smoking status

|  | **Overall cohort** | | **Sample included in model adjusting for potential confounders** | | **Individuals with missing Carstairs data** | | **Sample included in model adjusting for potential mediators** | | **Individuals with missing BMI data** | | **Individuals with missing smoking data** | |
| --- | --- | --- | --- | --- | --- | --- | --- | --- | --- | --- | --- | --- |
|  | **With atopic eczema** | **Without atopic eczema** | **With atopic eczema** | **Without atopic eczema** | **With atopic eczema** | **Without atopic eczema** | **With atopic eczema** | **Without atopic eczema** | **With atopic eczema** | **Without atopic eczema** | **With atopic eczema** | **Without atopic eczema** |
| **Number** | 215,073 | 646,539 | 212,500 | 639,470 | 2,573 | 7,069 | 170,780 | 454,950 | 34,228 | 126,122 | 3,920 | 21,463 |
| **Follow-up ^a^** |  |  |  |  |  |  |  |  |  |  |  |  |
| Total person-years | 845,534 | 2,381,779 | 833,785 | 2,350,950 | 11,749 | 30,828 | 697,799 | 1,765,185 | 109,802 | 393,188 | 7,211 | 47,706 |
| Median (IQR) duration of follow-up (years) | 3.2 (1.3-5.9) | 2.8 (1.2-5.5) | 3.2 (1.3-5.9) | 2.8 (1.2-5.5) | 4.0 (1.6-7.0) | 3.6 (1.5-6.6) | 3.3 (1.4-6.2) | 3.1 (1.3-5.8) | 2.4 (1.0-4.7) | 2.3 (0.9-4.6) | 1.4 (0.5-2.5) | 1.5 (0.5-3.1) |
| **Sex** |  |  |  |  |  |  |  |  |  |  |  |  |
| Female (%) | 119,149 (55.4%) | 354,208 (54.8%) | 117,663 (55.4%) | 350,103 (54.7%) | 1,486 (57.8%) | 4,105 (58.1%) | 100,514 (58.9%) | 269,245 (59.2%) | 13,709 (40.1%) | 52,617 (41.7%) | 1,319 (33.6%) | 7,385 (34.4%) |
| **Age (years) ^b^** |  |  |  |  |  |  |  |  |  |  |  |  |
| 18-29 | 83,066 (38.6%) | 267,060 (41.3%) | 82,012 (38.6%) | 264,086 (41.3%) | 1,054 (41.0%) | 2,974 (42.1%) | 51,974 (30.4%) | 136,698 (30.0%) | 25,495 (74.5%) | 89,354 (70.8%) | 3,539 (90.3%) | 17,269 (80.5%) |
| 30-39 | 36,715 (17.1%) | 113,881 (17.6%) | 36,122 (17.0%) | 112,161 (17.5%) | 593 (23.0%) | 1,720 (24.3%) | 31,578 (18.5%) | 88,340 (19.4%) | 3,589 (10.5%) | 15,735 (12.5%) | 164 (4.2%) | 1,888 (8.8%) |
| 40-49 | 25,545 (11.9%) | 70,699 (10.9%) | 25,234 (11.9%) | 69,899 (10.9%) | 311 (12.1%) | 800 (11.3%) | 22,945 (13.4%) | 58,979 (13.0%) | 1,705 (5.0%) | 7,052 (5.6%) | 70 (1.8%) | 890 (4.1%) |
| 50-59 | 21,605 (10.0%) | 59,361 (9.2%) | 21,397 (10.1%) | 58,833 (9.2%) | 208 (8.1%) | 528 (7.5%) | 19,957 (11.7%) | 51,726 (11.4%) | 1,023 (3.0%) | 4,505 (3.6%) | 48 (1.2%) | 620 (2.9%) |
| 60-69 | 21,999 (10.2%) | 63,035 (9.7%) | 21,837 (10.3%) | 62,606 (9.8%) | 162 (6.3%) | 429 (6.1%) | 20,680 (12.1%) | 56,724 (12.5%) | 867 (2.5%) | 3,509 (2.8%) | 42 (1.1%) | 397 (1.8%) |
| 70+ | 26,143 (12.2%) | 72,503 (11.2%) | 25,898 (12.2%) | 71,885 (11.2%) | 245 (9.5%) | 618 (8.7%) | 23,646 (13.8%) | 62,483 (13.7%) | 1,549 (4.5%) | 5,967 (4.7%) | 57 (1.5%) | 399 (1.9%) |
| **Ethnicity** |  |  |  |  |  |  |  |  |  |  |  |  |
| White | 183,612 (85.4%) | 548,100 (84.8%) | 181,173 (85.3%) | 541,478 (84.7%) | 2,439 (94.8%) | 6,622 (93.7%) | 145,302 (85.1%) | 385,813 (84.8%) | 29,239 (85.4%) | 106,394 (84.4%) | 3,300 (84.2%) | 17,453 (81.3%) |
| Minority ethnic | 31,461 (14.6%) | 98,439 (15.2%) | 31,327 (14.7%) | 97,992 (15.3%) | 134 (5.2%) | 447 (6.3%) | 25,478 (14.9%) | 69,137 (15.2%) | 4,989 (14.6%) | 19,728 (15.6%) | 620 (15.8%) | 4,010 (18.7%) |
| **Quintiles of Carstairs deprivation index ^c^** |  |  |  |  |  |  |  |  |  |  |  |  |
| 1 - Least deprived | 40,005 (18.6%) | 112,671 (17.4%) | 40,005 (18.8%) | 112,671 (17.6%) | n/a | n/a | 32,831 (19.2%) | 82,441 (18.1%) | 5,801 (16.9%) | 20,552 (16.3%) | 681 (17.4%) | 3,460 (16.1%) |
| 2 | 42,331 (19.7%) | 127,095 (19.7%) | 42,331 (19.9%) | 127,095 (19.9%) | n/a | n/a | 34,246 (20.1%) | 92,434 (20.3%) | 6,540 (19.1%) | 23,280 (18.5%) | 772 (19.7%) | 3,783 (17.6%) |
| 3 | 44,016 (20.5%) | 129,585 (20.0%) | 44,016 (20.7%) | 129,585 (20.3%) | n/a | n/a | 35,662 (20.9%) | 93,125 (20.5%) | 6,755 (19.7%) | 24,451 (19.4%) | 800 (20.4%) | 4,325 (20.2%) |
| 4 | 45,293 (21.1%) | 138,247 (21.4%) | 45,293 (21.3%) | 138,247 (21.6%) | n/a | n/a | 35,968 (21.1%) | 96,804 (21.3%) | 7,626 (22.3%) | 28,298 (22.4%) | 814 (20.8%) | 4,633 (21.6%) |
| 5 - Most deprived | 40,855 (19.0%) | 131,872 (20.4%) | 40,855 (19.2%) | 131,872 (20.6%) | n/a | n/a | 32,073 (18.8%) | 90,146 (19.8%) | 7,296 (21.3%) | 28,742 (22.8%) | 842 (21.5%) | 5,098 (23.8%) |
| Missing | 2,573 (1.2%) | 7,069 (1.1%) | n/a | n/a | n/a | n/a | n/a | n/a | 210 (0.6%) | 799 (0.6%) | 11 (0.3%) | 164 (0.8%) |
| **Body mass index (kg/m^2^) ^d^** |  |  |  |  |  |  |  |  |  |  |  |  |
| Underweight (<18.5) | 5,583 (2.6%) | 18,965 (2.9%) | 5,525 (2.6%) | 18,766 (2.9%) | 58 (2.3%) | 199 (2.8%) | 5,040 (3.0%) | 14,997 (3.3%) | n/a | n/a | 52 (1.3%) | 260 (1.2%) |
| Normal (18.5-24.9) | 78,647 (36.6%) | 233,286 (36.1%) | 77,544 (36.5%) | 230,252 (36.0%) | 1,103 (42.9%) | 3,034 (42.9%) | 73,849 (43.2%) | 199,864 (43.9%) | n/a | n/a | 276 (7.0%) | 1,377 (6.4%) |
| Overweight (25-29.9) | 57,766 (26.9%) | 163,116 (25.2%) | 57,035 (26.8%) | 161,216 (25.2%) | 731 (28.4%) | 1,900 (26.9%) | 54,961 (32.2%) | 145,942 (32.1%) | n/a | n/a | 110 (2.8%) | 604 (2.8%) |
| Obese (30+) | 38,849 (18.1%) | 105,050 (16.2%) | 38,378 (18.1%) | 103,913 (16.2%) | 471 (18.3%) | 1,137 (16.1%) | 36,930 (21.6%) | 94,147 (20.7%) | n/a | n/a | 98 (2.5%) | 380 (1.8%) |
| Missing | 34,228 (15.9%) | 126,122 (19.5%) | 34,018 (16.0%) | 125,323 (19.6%) | 210 ( 8.2%) | 799 (11.3%) | n/a | n/a | n/a | n/a | 3,384 (86.3%) | 18,842 (87.8%) |
| **Smoking status ^d^** |  |  |  |  |  |  |  |  |  |  |  |  |
| Non-smoker | 115,457 (53.7%) | 346,669 (53.6%) | 114,066 (53.7%) | 342,819 (53.6%) | 1,391 (54.1%) | 3,850 (54.5%) | 89,109 (52.2%) | 239,210 (52.6%) | 20,810 (60.8%) | 70,632 (56.0%) | n/a | n/a |
| Current or ex-smoker | 95,696 (44.5%) | 278,407 (43.1%) | 94,525 (44.5%) | 275,352 (43.1%) | 1,171 (45.5%) | 3,055 (43.2%) | 81,671 (47.8%) | 215,740 (47.4%) | 10,034 (29.3%) | 36,648 (29.1%) | n/a | n/a |
| Missing | 3,920 (1.8%) | 21,463 (3.3%) | 3,909 (1.8%) | 21,299 (3.3%) | 11 (0.4%) | 164 (2.3%) | n/a | n/a | 3,384 (9.9%) | 18,842 (14.9%) | n/a | n/a |
| **Charlson Comorbidity Index ^d^** |  |  |  |  |  |  |  |  |  |  |  |  |
| Low (0) | 134,391 (62.5%) | 479,693 (74.2%) | 132,686 (62.4%) | 474,241 (74.2%) | 1,705 (66.3%) | 5,452 (77.1%) | 105,375 (61.7%) | 326,174 (71.7%) | 23,165 (67.7%) | 103,962 (82.4%) | 3,277 (83.6%) | 19,614 (91.4%) |
| Moderate (1-2) | 71,488 (33.2%) | 139,122 (21.5%) | 70,731 (33.3%) | 137,779 (21.5%) | 757 (29.4%) | 1,343 (19.0%) | 57,128 (33.5%) | 104,644 (23.0%) | 10,506 (30.7%) | 20,305 (16.1%) | 617 (15.7%) | 1,764 (8.2%) |
| Severe (3 or more) | 9,194 (4.3%) | 27,724 (4.3%) | 9,083 (4.3%) | 27,450 (4.3%) | 111 (4.3%) | 274 (3.9%) | 8,277 (4.8%) | 24,132 (5.3%) | 557 (1.6%) | 1,855 (1.5%) | 26 (0.7%) | 85 (0.4%) |
| **Asthma (%) ^d^** | 54,774 (25.5%) | 82,629 (12.8%) | 54,194 (25.5%) | 81,832 (12.8%) | 580 (22.5%) | 797 (11.3%) | 42,587 (24.9%) | 57,705 (12.7%) | 8,921 (26.1%) | 14,688 (11.6%) | 458 (11.7%) | 1,208 (5.6%) |
| **Harmful alcohol use (%) ^d^** | 15,943 (7.4%) | 40,279 (6.2%) | 15,733 (7.4%) | 39,803 (6.2%) | 210 (8.2%) | 476 (6.7%) | 14,377 (8.4%) | 33,805 (7.4%) | 930 (2.7%) | 2,962 (2.3%) | 53 (1.4%) | 215 1.0%) |
| **Problems with sleep (%) ^d^** | 37,355 (17.4%) | 66,682 (10.3%) | 36,757 (17.3%) | 65,742 (10.3%) | 598 (23.2%) | 940 (13.3%) | 30,521 (17.9%) | 50,775 (11.2%) | 4,939 (14.4%) | 9,191 (7.3%) | 365 (9.3%) | 793 (3.7%) |

Abbreviations: IQR: Interquartile range

Individuals can contribute data as both atopic eczema exposed and unexposed. Therefore, numbers of exposed/unexposed do not total the whole cohort, as individuals may be included in more than one column.

^a^ Follow-up based on censoring at the earliest of: death, no longer registered with practice, practice no longer contributing to CPRD, or depression or anxiety diagnosis, diagnosis that suggests an alternative cause of the depression or anxiety outcome (severe mental illness)

^b^ Age at index date

^c^ Carstairs deprivation index based on practice-level data (from 2011).

^d^ Based on records closest to index date.

|  | | |  | |  |  |  |  |  |  |
| --- | --- | --- | --- | --- | --- | --- | --- | --- | --- | --- |
|  |  |  | |  |  |  |  |  |  |  |
|  | | |  | |  |  |  |  |  |  |
|  | | |  | |  |  |  |  |  |  |
|  | | |  | |  |  |  |  |  |  |

Table S8: Characteristics of the anxiety cohort at cohort entry, for: the overall cohort, individuals included in the model additionally adjusting for potential confounders (i.e., individuals with no missing Carstairs deprivation data), individuals with missing Carstairs data, individuals included in the model additionally adjusting for potential mediators (i.e., individuals with no missing BMI or smoking status data), and for individuals with missing BMI or smoking status

|  | **Overall cohort** | | **Sample included in model adjusting for potential confounders** | | **Individuals with missing Carstairs data** | | **Sample included in model adjusting for potential mediators** | | **Individuals with missing BMI data** | | **Individuals with missing smoking data** | |
| --- | --- | --- | --- | --- | --- | --- | --- | --- | --- | --- | --- | --- |
|  | **With atopic eczema** | **Without atopic eczema** | **With atopic eczema** | **Without atopic eczema** | **With atopic eczema** | **Without atopic eczema** | **With atopic eczema** | **Without atopic eczema** | **With atopic eczema** | **Without atopic eczema** | **With atopic eczema** | **Without atopic eczema** |
| **Number** | 242,598 | 774,113 | 239,774 | 765,991 | 2,824 | 8,122 | 196,307 | 560,318 | 36,120 | 139,386 | 3,950 | 22,578 |
| **Follow-up ^a^** |  |  |  |  |  |  |  |  |  |  |  |  |
| Total person-years | 970,230 | 2,898,227 | 957,184 | 2,861,456 | 13,045 | 36,771 | 813,597 | 2,199,809 | 117,507 | 438,007 | 7,319 | 50,928 |
| Median (IQR) duration of follow-up (years) | 3.3 (1.4-6.0) | 2.9 (1.2-5.6) | 3.2 (1.4-6.0) | 2.9 (1.2-5.6) | 4.0 (1.7-7.1) | 3.8 (1.5-6.9) | 3.4 (1.5-6.2) | 3.1 (1.3-5.9) | 2.4 (1.0-4.8) | 2.3 (0.9-4.6) | 1.4 (0.5-2.5) | 1.5 (0.5-3.2) |
| **Sex** |  |  |  |  |  |  |  |  |  |  |  |  |
| Female (%) | 138,964 (57.3%) | 446,169 (57.6%) | 137,304 (57.3%) | 441,348 (57.6%) | 1,660 (58.8%) | 4,821 (59.4%) | 118,986 (60.6%) | 346,307 (61.8%) | 14,990 (41.5%) | 61,391 (44.0%) | 1,330 (33.7%) | 8,046 (35.6%) |
| **Age (years) ^b^** |  |  |  |  |  |  |  |  |  |  |  |  |
| 18-29 | 88,755 (36.6%) | 297,014 (38.4%) | 87,674 (36.6%) | 293,892 (38.4%) | 1,081 (38.3%) | 3,122 (38.4%) | 57,191 (29.1%) | 159,020 (28.4%) | 26,035 (72.1%) | 94,057 (67.5%) | 3,524 (89.2%) | 17,633 (78.1%) |
| 30-39 | 42,298 (17.4%) | 140,665 (18.2%) | 41,633 (17.4%) | 138,644 (18.1%) | 665 (23.5%) | 2,021 (24.9%) | 36,638 (18.7%) | 109,720 (19.6%) | 4,086 (11.3%) | 18,977 (13.6%) | 178 (4.5%) | 2,164 (9.6%) |
| 40-49 | 31,182 (12.9%) | 94,977 (12.3%) | 30,814 (12.9%) | 93,953 (12.3%) | 368 (13.0%) | 1,024 (12.6%) | 28,250 (14.4%) | 79,652 (14.2%) | 2,021 (5.6%) | 9,170 (6.6%) | 75 (1.9%) | 1,104 (4.9%) |
| 50-59 | 26,090 (10.8%) | 78,533 (10.1%) | 25,845 (10.8%) | 77,856 (10.2%) | 245 (8.7%) | 677 (8.3%) | 24,276 (12.4%) | 68,924 (12.3%) | 1,185 (3.3%) | 5,650 (4.1%) | 54 (1.4%) | 720 (3.2%) |
| 60-69 | 25,257 (10.4%) | 77,543 (10.0%) | 25,056 (10.4%) | 76,975 (10.0%) | 201 (7.1%) | 568 (7.0%) | 23,775 (12.1%) | 69,848 (12.5%) | 987 (2.7%) | 4,238 (3.0%) | 46 (1.2%) | 482 (2.1%) |
| 70+ | 29,016 (12.0%) | 85,381 (11.0%) | 28,752 (12.0%) | 84,671 (11.1%) | 264 (9.3%) | 710 (8.7%) | 26,177 (13.3%) | 73,154 (13.1%) | 1,806 (5.0%) | 7,294 (5.2%) | 73 (1.8%) | 475 (2.1%) |
| **Ethnicity** |  |  |  |  |  |  |  |  |  |  |  |  |
| White | 208,462 (85.9%) | 661,005 (85.4%) | 205,779 (85.8%) | 653,373 (85.3%) | 2,683 (95.0%) | 7,632 (94.0%) | 168,344 (85.8%) | 478,957 (85.5%) | 30,933 (85.6%) | 118,010 (84.7%) | 3,326 (84.2%) | 18,384 (81.4%) |
| Minority ethnic | 34,136 (14.1%) | 113,108 (14.6%) | 33,995 (14.2%) | 112,618 (14.7%) | 141 (5.0%) | 490 (6.0%) | 27,963 (14.2%) | 81,361 (14.5%) | 5,187 (14.4%) | 21,376 (15.3%) | 624 (15.8%) | 4,194 (18.6%) |
| **Quintiles of Carstairs deprivation index ^c^** |  |  |  |  |  |  |  |  |  |  |  |  |
| 1 - Least deprived | 44,123 (18.2%) | 132,325 (17.1%) | 44,123 (18.4%) | 132,325 (17.3%) | n/a | n/a | 36,642 (18.7%) | 99,085 (17.7%) | 6,105 (16.9%) | 22,487 (16.1%) | 684 (17.3%) | 3,629 (16.1%) |
| 2 | 46,993 (19.4%) | 149,716 (19.3%) | 46,993 (19.6%) | 149,716 (19.5%) | n/a | n/a | 38,685 (19.7%) | 111,359 (19.9%) | 6,850 (19.0%) | 25,543 (18.3%) | 774 (19.6%) | 3,932 (17.4%) |
| 3 | 49,443 (20.4%) | 154,764 (20.0%) | 49,443 (20.6%) | 154,764 (20.2%) | n/a | n/a | 40,768 (20.8%) | 114,151 (20.4%) | 7,092 (19.6%) | 27,004 (19.4%) | 807 (20.4%) | 4,586 (20.3%) |
| 4 | 52,153 (21.5%) | 168,516 (21.8%) | 52,153 (21.8%) | 168,516 (22.0%) | n/a | n/a | 42,403 (21.6%) | 121,913 (21.8%) | 8,091 (22.4%) | 31,517 (22.6%) | 842 (21.3%) | 4,918 (21.8%) |
| 5 - Most deprived | 47,062 (19.4%) | 160,670 (20.8%) | 47,062 (19.6%) | 160,670 (21.0%) | n/a | n/a | 37,809 (19.3%) | 113,810 (20.3%) | 7,764 (21.5%) | 31,973 (22.9%) | 830 (21.0%) | 5,342 (23.7%) |
| Missing | 2,824 (1.2%) | 8,122 (1.0%) | n/a | n/a | n/a | n/a | n/a | n/a | 218 (0.6%) | 862 (0.6%) | 13 (0.3%) | 171 (0.8%) |
| **Body mass index (kg/m^2^) ^d^** |  |  |  |  |  |  |  |  |  |  |  |  |
| Underweight (<18.5) | 6,156 (2.5%) | 22,161 (2.9%) | 6,093 (2.5%) | 21,951 (2.9%) | 63 (2.2%) | 210 (2.6%) | 5,628 (2.9%) | 17,890 (3.2%) | n/a | n/a | 56 (1.4%) | 274 (1.2%) |
| Normal (18.5-24.9) | 87,042 (35.9%) | 276,952 (35.8%) | 85,862 (35.8%) | 273,573 (35.7%) | 1,180 (41.8%) | 3,379 (41.6%) | 82,353 (42.0%) | 239,901 (42.8%) | n/a | n/a | 285 (7.2%) | 1,509 (6.7%) |
| Overweight (25-29.9) | 65,734 (27.1%) | 198,662 (25.7%) | 64,917 (27.1%) | 196,450 (25.6%) | 817 (28.9%) | 2,212 (27.2%) | 62,869 (32.0%) | 178,900 (31.9%) | n/a | n/a | 119 (3.0%) | 691 (3.1%) |
| Obese (30+) | 47,546 (19.6%) | 136,952 (17.7%) | 47,000 (19.6%) | 135,493 (17.7%) | 546 (19.3%) | 1,459 (18.0%) | 45,457 (23.2%) | 123,627 (22.1%) | n/a | n/a | 112 (2.8%) | 422 (1.9%) |
| Missing | 36,120 (14.9%) | 139,386 (18.0%) | 35,902 (15.0%) | 138,524 (18.1%) | 218 (7.7%) | 862 (10.6%) | n/a | n/a | n/a | n/a | 3,378 (85.5%) | 19,682 (87.2%) |
| **Smoking status ^d^** |  |  |  |  |  |  |  |  |  |  |  |  |
| Non-smoker | 124,789 (51.4%) | 399,346 (51.6%) | 123,333 (51.4%) | 395,175 (51.6%) | 1,456 (51.6%) | 4,171 (51.4%) | 97,963 (49.9%) | 283,095 (50.5%) | 21,427 (59.3%) | 76,157 (54.6%) | n/a | n/a |
| Current or ex-smoker | 113,859 (46.9%) | 352,189 (45.5%) | 112,504 (46.9%) | 348,409 (45.5%) | 1,355 (48.0%) | 3,780 (46.5%) | 98,344 (50.1%) | 277,223 (49.5%) | 11,315 (31.3%) | 43,547 (31.2%) | n/a | n/a |
| Missing | 3,950 (1.6%) | 22,578 (2.9%) | 3,937 (1.6%) | 22,407 (2.9%) | 13 (0.5%) | 171 (2.1%) | n/a | n/a | 3,378 (9.4%) | 19,682 (14.1%) | n/a | n/a |
| **Charlson Comorbidity Index ^d^** |  |  |  |  |  |  |  |  |  |  |  |  |
| Low (0) | 149,031 (61.4%) | 565,797 (73.1%) | 147,185 (61.4%) | 559,610 (73.1%) | 1,846 (65.4%) | 6,187 (76.2%) | 118,905 (60.6%) | 396,247 (70.7%) | 24,252 (67.1%) | 113,953 (81.8%) | 3,290 (83.3%) | 20,613 (91.3%) |
| Moderate (1-2) | 82,401 (34.0%) | 172,496 (22.3%) | 81,550 (34.0%) | 170,920 (22.3%) | 851 (30.1%) | 1,576 (19.4%) | 67,329 (34.3%) | 132,995 (23.7%) | 11,186 (31.0%) | 22,941 (16.5%) | 626 (15.8%) | 1,871 (8.3%) |
| Severe (3 or more) | 11,166 (4.6%) | 35,820 (4.6%) | 11,039 (4.6%) | 35,461 (4.6%) | 127 (4.5%) | 359 (4.4%) | 10,073 (5.1%) | 31,076 (5.5%) | 682 (1.9%) | 2,492 (1.8%) | 34 (0.9%) | 94 (0.4%) |
| **Asthma (%) ^d^** | 63,142 (26.0%) | 102,633 (13.3%) | 62,501 (26.1%) | 101,698 (13.3%) | 641 (22.7%) | 935 (11.5%) | 50,437 (25.7%) | 74,744 (13.3%) | 9,442 (26.1%) | 16,158 (11.6%) | 467 (11.8%) | 1,234 (5.5%) |
| **Harmful alcohol use (%) ^d^** | 19,740 (8.1%) | 53,118 (6.9%) | 19,484 (8.1%) | 52,503 (6.9%) | 256 (9.1%) | 615 (7.6%) | 17,890 (9.1%) | 44,882 (8.0%) | 1,150 (3.2%) | 3,854 (2.8%) | 62 (1.6%) | 271 (1.2%) |
| **Problems with sleep (%) ^d^** | 47,582 (19.6%) | 93,110 (12.0%) | 46,876 (19.6%) | 91,862 (12.0%) | 706 (25.0%) | 1,248 (15.4%) | 39,944 (20.3%) | 73,060 (13.0%) | 5,531 (15.3%) | 11,278 (8.1%) | 389 (9.8%) | 850 (3.8%) |

Abbreviations: IQR: Interquartile range

Individuals can contribute data as both atopic eczema exposed and unexposed. Therefore, numbers of exposed/unexposed do not total the whole cohort, as individuals may be included in more than one column.

^a^ Follow-up based on censoring at the earliest of: death, no longer registered with practice, practice no longer contributing to CPRD, or depression or anxiety diagnosis, diagnosis that suggests an alternative cause of the depression or anxiety outcome (severe mental illness)

^b^ Age at index date

^c^ Carstairs deprivation index based on practice-level data (from 2011).

^d^ Based on records closest to index date.

|  | | |  | |  |  |  |  |  |  |
| --- | --- | --- | --- | --- | --- | --- | --- | --- | --- | --- |
|  |  |  | |  |  |  |  |  |  |  |
|  | | |  | |  |  |  |  |  |  |
|  | | |  | |  |  |  |  |  |  |
|  | | |  | |  |  |  |  |  |  |

Table S9: HRs (95% CI) ^a^ for the association between atopic eczema and depression or anxiety. Fitted to adults with complete data for all variables included in each model and from valid matched sets ^b^

| **Cohort** | **Minimally adjusted ^c^** | | | **Further adjusted for potential confounders ^d^** | | | **Additionally adjusted for potential mediators ^e^** | | |
| --- | --- | --- | --- | --- | --- | --- | --- | --- | --- |
|  | **Number** | **Events/PYAR** | **HR (95% CI)** | **Number** | **Events/PYAR** | **HR (95% CI)** | **Number** | **Events/PYAR** | **HR (95% CI)** |
| **Depression** |  |  |  |  |  |  |  |  |  |
| Without atopic eczema | 646,539 | 46,503/2,381,779 | 1 (reference) | 639,470 | 45,833/2,350,951 | 1 (reference) | 454,950 | 34,140/1,765,185 | 1 (reference) |
| With atopic eczema | 215,073 | 19,481/845,534 | 1.17 (1.14,1.19) | 212,500 | 19,165/833,785 | 1.17 (1.14,1.19) | 170,780 | 16,101/697,8900 | 1.06 (1.03,1.08) |
| **Anxiety** |  |  |  |  |  |  |  |  |  |
| Without atopic eczema | 774,113 | 41,670/2,898,227 | 1 (reference) | 765,991 | 40,996/2,861,456 | 1 (reference) | 560,318 | 31,488/2,199,809 | 1 (reference) |
| With atopic eczema | 242,598 | 16,893/970,230 | 1.19 (1.17,1.21) | 239,774 | 16,581/957,185 | 1.19 (1.16,1.21) | 196,307 | 14,171/813,598 | 1.08 (1.06,1.11) |

Abbreviations: CI – Confidence Interval; HR – Hazard Ratio; PYAR – Person years at risk

^a^ Estimated hazard ratios from Cox regression with current age as underlying timescale, stratified by matched set (matched on age at cohort entry, sex, general practice, and date at cohort entry)

^b^ Matched sets including one individual with atopic eczema and at least one matched comparator without.

^c^ Adjusted for matching variables (age, sex, practice)

^d^ Minimally adjusted model further adjusted for calendar period and deprivation (using quintiles of Carstairs deprivation index [using 2011 census data)])

^e^ Cohort is further adjusted for comorbidity burden (using the Charlson comorbidity index), comorbid asthma, sleep problems, smoking status, high dose glucocorticoid use, harmful alcohol use and body mass index

| **Cohort** | **Total PYAR at each level of atopic eczema severity** | | | **Total follow-up** |
| --- | --- | --- | --- | --- |
|  | **Mild eczema** | **Moderate eczema** | **Severe eczema** |  |
| **Depression** |  |  |  |  |
| *White* | 570,467 (77.6) | 155,585 (21.2) | 9,443 (1.2) | 735,495 |
| *Minority ethnic* | 85,437 (77.7) | 22,938 (20.8) | 1,664 (1.5) | 110,039 |
| **Anxiety** |  |  |  |  |
| *White* | 655,936 (77.2) | 182,098 (21.5) | 10,862 (1.3) | 848,896 |
| *Minority ethnic* | 93681 (77.2) | 25819 (21.3) | 1834 (1.5) | 121,334 |

Table S10: Proportions of total follow up each ethnic group (white or minority ethnic) spends at each level of atopic eczema (mild, moderate, or severe) severity during follow up. Data are n (%)

Abbreviations: PYAR – Person years at risk

Table S11: HRs (95% CI) for the association between atopic eczema severity and depression or anxiety in white and minority ethnic groups

| **Cohort** | **Minimally adjusted** | | | **Confounder adjusted** | | | **Mediator adjusted** | | |
| --- | --- | --- | --- | --- | --- | --- | --- | --- | --- |
|  | **Number** | **Events/PYAR** | **HR (95% CI)** | **Number** | **Events/PYAR** | **HR (95% CI)** | **Number** | **Events/PYAR** | **HR (95% CI)** |
| **Depression** |  |  |  |  |  |  |  |  |  |
| *White* |  |  |  |  |  |  |  |  |  |
| Unexposed | 548,100 | 42,656/2,083,588 | 1 (reference) | 541,478 | 41,994/2,054,242 | 1 (reference) | 385,813 | 31,151/1,542,760 | 1 (reference) |
| Mild | 152,503 | 13,208/570,467 | 1.09 (1.06, 1.11) | 150,521 | 12,996/562,033 | 1.09 (1.06, 1.11) | 118,859 | 10,760/462,020 | 1.01 (0.98, 1.03) |
| Moderate | 32,616 | 4,095/155,585 | 1.39 (1.33, 1.45) | 32,131 | 4,001/153,088 | 1.38 (1.33, 1.45) | 27,841 | 3,461/134,754 | 1.22 (1.16, 1.28) |
| Severe | 2,076 | 252/9,443 | 1.31 (1.10, 1.55) | 2,042 | 249/9,221 | 1.38 (1.15, 1.64) | 1,743 | 215/7,993 | 1.27 (1.04, 1.56) |
| *Minority ethnic* |  |  |  |  |  |  |  |  |  |
| Unexposed | 98,439 | 3,847/298,191 | 1 (reference) | 97,992 | 3,839/296,708 | 1 (reference) | 69,137 | 2,989/222,425 | 1 (reference) |
| Mild | 26,426 | 1,400/85,437 | 1.19 (1.07, 1.31) | 26,317 | 1,395/84,974 | 1.20 (1.08, 1.32) | 21,098 | 1,202/71,247 | 1.01 (0.90, 1.14) |
| Moderate | 5,092 | 496/22,938 | 1.91 (1.59, 2.30) | 5,068 | 494/22,811 | 1.90 (1.58, 2.29) | 4,450 | 438/20,367 | 1.71 (1.37, 2.14) |
| Severe | 386 | 30/1,665 | 1.55 (0.76, 3.15) | 385 | 30/1,657 | 1.60 (0.79, 3.26) | 326 | 25/1,419 | 1.40 (0.61, 3.21) |
| **Anxiety** |  |  |  |  |  |  |  |  |  |
| *White* |  |  |  |  |  |  |  |  |  |
| Unexposed | 661,005 | 38,443/2,550,372 | 1 (reference) | 653,373 | 37,781/2,515,225 | 1 (reference) | 478,957 | 29,019/1,934,703 | 1 (reference) |
| Mild | 172,887 | 11,569/655,937 | 1.10 (1.08, 1.13) | 170,721 | 11,359/646,642 | 1.10 (1.07, 1.13) | 137,741 | 9,606/541,356 | 1.02 (0.99, 1.05) |
| Moderate | 37,552 | 3,593/182,098 | 1.43 (1.37, 1.50) | 36,998 | 3,503/179,234 | 1.43 (1.36, 1.50) | 32,444 | 3,050/159,473 | 1.25 (1.18, 1.32) |
| Severe | 2,350 | 222/10,862 | 1.34 (1.12, 1.61) | 2,312 | 218/10,608 | 1.34 (1.11, 1.60) | 1,992 | 193/9,248 | 1.21 (0.98, 1.50) |
| *Minority ethnic* |  |  |  |  |  |  |  |  |  |
| Unexposed | 113,108 | 3,227/347,855 | 1 (reference) | 112,618 | 3,215/346,231 | 1 (reference) | 81,361 | 2,469/265,107 | 1 (reference) |
| Mild | 28,591 | 1,096/93,681 | 1.30 (1.17, 1.46) | 28,476 | 1,090/93,193 | 1.32 (1.18, 1.47) | 23,113 | 955/78,907 | 1.16 (1.02, 1.33) |
| Moderate | 5,632 | 383/25,819 | 1.83 (1.49, 2.25) | 5,607 | 381/25,683 | 1.83 (1.49, 2.25) | 4,948 | 344/23,064 | 1.49 (1.18, 1.89) |
| Severe | 420 | 30/1,834 | 1.15 (0.47, 2.84) | 419 | 30/1,826 | 1.11 (0.45, 2.75) | 356 | 23/1,551 | 0.45 (0.14, 1.51) |

P values for interaction by ethnicity is p<0.01 for all models and outcomes. Abbreviations: CI – Confidence Interval; HR – Hazard Ratio; PYAR – Person years at risk

^a^ Matched sets including one exposed patient and at least one unexposed patient.

^b^ Adjusted for calendar time and quintiles of Carstairs deprivation index (using 2011 census data)

^c^ Cohort is further adjusted for comorbidity burden (using the Charlson comorbidity index), comorbid asthma, sleep problems, smoking status, high dose glucocorticoid use, harmful alcohol use and body mass index

^d^ Estimated hazard ratios from Cox regression with current age as underlying timescale, stratified by matched set (matched on age at cohort entry, sex, general practice, and date at cohort entry)
